# Potential effects of disruption to HIV programmes in sub-Saharan Africa caused by COVID-19: results from multiple mathematical models

**DOI:** 10.1016/S2352-3018(20)30211-3

**Published:** 2020-08-06

**Authors:** Britta L Jewell, Edinah Mudimu, John Stover, Debra ten Brink, Andrew N Phillips, Jennifer A Smith, Rowan Martin-Hughes, Yu Teng, Robert Glaubius, Severin Guy Mahiane, Loveleen Bansi-Matharu, Isaac Taramusi, Newton Chagoma, Michelle Morrison, Meg Doherty, Kimberly Marsh, Anna Bershteyn, Timothy B Hallett, Sherrie L Kelly

**Affiliations:** a Medical Research Council Centre for Global Infectious Disease Analysis, Abdul Latif Jameel Institute for Disease and Emergency Analytics, Imperial College London, London, UK; b Department of Decision Sciences, University of South Africa, Pretoria, South Africa; c Avenir Health, Glastonbury, CT, USA; d Burnet Institute, Melbourne, VIC, Australia; e Institute for Global Health, University College London, London, UK; f National AIDS Council of Zimbabwe, Harare, Zimbabwe; g National AIDS Council of Malawi, Lilongwe, Malawi; h Bill & Melinda Gates Foundation, Seattle, WA, USA; i WHO, Geneva, Switzerland; j UNAIDS, Geneva, Switzerland; k New York University School of Medicine, New York, NY, USA

## Abstract

**Background:**

The COVID-19 pandemic could lead to disruptions to provision of HIV services for people living with HIV and those at risk of acquiring HIV in sub-Saharan Africa, where UNAIDS estimated that more than two-thirds of the approximately 38 million people living with HIV resided in 2018. We aimed to predict the potential effects of such disruptions on HIV-related deaths and new infections in sub-Saharan Africa.

**Methods:**

In this modelling study, we used five well described models of HIV epidemics (Goals, Optima HIV, HIV Synthesis, an Imperial College London model, and Epidemiological MODeling software [EMOD]) to estimate the effect of various potential disruptions to HIV prevention, testing, and treatment services on HIV-related deaths and new infections in sub-Saharan Africa lasting 6 months over 1 year from April 1, 2020. We considered scenarios in which disruptions affected 20%, 50%, and 100% of the population.

**Findings:**

A 6-month interruption of supply of antiretroviral therapy (ART) drugs across 50% of the population of people living with HIV who are on treatment would be expected to lead to a 1·63 times (median across models; range 1·39–1·87) increase in HIV-related deaths over a 1-year period compared with no disruption. In sub-Saharan Africa, this increase amounts to a median excess of HIV deaths, across all model estimates, of 296 000 (range 229 023–420 000) if such a high level of disruption occurred. Interruption of ART would increase mother-to-child transmission of HIV by approximately 1·6 times. Although an interruption in the supply of ART drugs would have the largest impact of any potential disruptions, effects of poorer clinical care due to overstretched health facilities, interruptions of supply of other drugs such as co-trimoxazole, and suspension of HIV testing would all have a substantial effect on population-level mortality (up to a 1·06 times increase in HIV-related deaths over a 1-year period due to disruptions affecting 50% of the population compared with no disruption). Interruption to condom supplies and peer education would make populations more susceptible to increases in HIV incidence, although physical distancing measures could lead to reductions in risky sexual behaviour (up to 1·19 times increase in new HIV infections over a 1-year period if 50% of people are affected).

**Interpretation:**

During the COVID-19 pandemic, the primary priority for governments, donors, suppliers, and communities should focus on maintaining uninterrupted supply of ART drugs for people with HIV to avoid additional HIV-related deaths. The provision of other HIV prevention measures is also important to prevent any increase in HIV incidence.

**Funding:**

Bill & Melinda Gates Foundation.

## Introduction

Disruption to delivery of health care in sub-Saharan African settings caused by COVID-19 could lead to adverse consequences for the health of people beyond those from COVID-19 itself.^1^, ^2^, ^3^ Causes of such disruption could include COVID-19-related morbidity and mortality, clinic closures or reduced service availability, and physical distancing and other measures put in place to combat the virus spread.

HIV remains highly prevalent in sub-Saharan Africa with over 25·7 million (uncertainty range 22·2–29·5) people estimated to be living with HIV in the region in 2018.^4^ Concern exists that possible disruptions in HIV programmes due to COVID-19^1^, ^2^, ^5^ could affect HIV-related mortality and new infections. Negative effects of the COVID-19 epidemic on access to health services have begun to emerge. A survey of people living with HIV run by the Human Sciences Research Council in South Africa via a social media platform found that 13% of people said they did not have access to their chronic medication during lockdown,^6^ with some reports in the area as of May, 2020, showing that only 30–50% of patients were collecting their medication.^7^ A rapid survey assessment in Zimbabwe in April, 2020, found 19% of people with HIV attempting to get a refill of an antiretroviral drug had not been able to, or were only able to get a partial refill,^8^ while a telephone-based survey in Kenya and Nigeria run by the Finmark Trust in April, 2020, found 14% of people were unable to collect needed medications.^9^ In a May, 2020, WHO survey in five of 13 countries in sub-Saharan Africa, antiretroviral therapy (ART) stock availability for major first-line drugs was reported to be 3 months or less, with reasons including failure of suppliers to deliver on time (Low-Beer D, WHO, Geneva, Switzerland, personal communication).

**Research in context**

**Evidence before this study**

The COVID-19 pandemic could lead to disruptions to provision of services for people with HIV in sub-Saharan Africa, but the relative consequences for HIV mortality and incidence of disruptions of different activities is not widely appreciated, and neither is the potential absolute magnitude of impact. We searched Web of Science on April 24, 2020, with no date or language restrictions, using the terms (COVID* AND model* AND HIV* AND Africa) and found no studies that predict the effects of disruption due to COVID-19 on HIV outcomes in sub-Saharan Africa.

**Added value of this study**

Our study provides a robust assessment of the potential effects of HIV service disruptions in sub-Saharan Africa resulting from COVID-19 and informs those organising programmes of the area of greatest priority, which is to maintain the supply of antiretroviral drugs for people with HIV. The consistency of the main findings across multiple models adds weight to the findings.

**Implications of all the available evidence**

During the COVID-19 pandemic, the primary priorities for governments, donors, suppliers, and communities to avoid additional HIV-related deaths and HIV incidence should be to maintain constant supply of antiretroviral drugs for people living with HIV. Provision of other HIV prevention interventions should also be carefully maintained to prevent an increase in HIV incidence.

We aimed to explore the effects of HIV service disruptions using existing mathematical models of HIV epidemiology and intervention programmes in sub-Saharan Africa. In this Article, we consider the predicted effects of temporary disruption to different individual HIV services for periods of 3 or 6 months over 1-year and 5-year periods on HIV mortality and incidence. We also considered disruptions that affect 20%, 50%, or 100% of the population. The primary analysis considered effects over 1 year of a 6-month disruption for 50% of the population.

## Methods

### Study design

In this modelling study, we used five well described existing HIV models to estimate the impact of different disruptions to HIV prevention and treatment services as a result of the COVID-19 pandemic on HIV-related mortality and incidence. These models were Goals,^10^ Optima HIV,^11^ HIV Synthesis,^12^ an Imperial College London Model,^13^ and Epidemiological MODeling software (EMOD).^14^ This study arose from discussions within an ad hoc group composed of the authors and other individuals (listed in the Acknowledgments).

### Models and data sources

The features of each model are summarised in table 1. Each model is based on information provided by a wide array of previous studies, as has been described previously.^10^, ^11^, ^12^, ^13^, ^14^ Importantly, the models make assumptions relating to effects of ART interruption that account for the fact that immune recovery during ART tends to be lost quite rapidly after interruption of treatment. These assumptions are based on, for example, empirical observations^15^, ^17^ that showed a median loss of 187 CD4 cells per μL after ART interruption and 25% of people have a loss of more than 317 cells per μL in 2 months.

**Table 1:** Characteristics of the contributing models

	**Goals**	**Optima HIV**	**HIV Synthesis**	**Imperial College London model**	**EMOD**
Structure	Compartment model with disaggregation by risk group	Population-based compartment model with sex, age, and risk group disaggregation; 1-month time step	Individual-based stochastic model; 3-month time step	Compartmental model with sex, age, and risk structure	Individual-based stochastic simulation; daily time step; network model
Approach to calibration of data and estimates for specific settings	Epidemiological parameters (probability of transmission per sex act; variation by stage of infection, presence of other STIs, and effectiveness of condoms and ART) are varied to fit the model to prevalence estimates from surveillance and surveys	Epidemiological parameters (probability of transmission per sex act, variation by stage of infection [informed by CD4 cell counts and viral load monitoring], HIV testing rate, mortality rate, presence of other ulcerative STIs or tuberculosis, or both, and effectiveness of condoms, circumcision, and unsuppressive or suppressive ART) are varied to fit the model to prevalence estimates from surveillance and surveys	Parameters relating to population characteristics, sexual behaviour (condomless sex), age-gender mixing (ie, distribution of ages of male sexual partners of women of a given age and vice versa), HIV acquisition, HIV testing, natural history (CD4 cell count and viral load), ART, and risk of AIDS and death varied within plausible bounds to create a range of setting scenarios; country situations are mimicked by selecting setting scenarios with epidemic and programmatic features consistent with country data	Epidemiological and HIV intervention parameters (probability of transmission per sex act, proportion of the population in each risk group, sex-specific and risk-specific sexual contact rates, risk-specific condom use, amount of mixing of at-risk groups, ART and VMMC uptake) are varied to fit the model to country-specific prevalence, incidence, ART and VMMC coverage, and mortality estimates, reported by UNAIDS	Parameterised with epidemiological data including population size, fertility, mortality, VMMC coverage, and health-seeking and sexual behaviour; data from South Africa on age-specific and sex-specific HIV prevalence, ART coverage, population size, and HIV incidence were used to calibrate the model; calibration was done using a parallel simultaneous perturbation optimisation algorithm; roulette resampling in proportion to the likelihood of each simulation was used to select 250 model parameter sets
Sexual behaviour	Behaviours (number of partners, sex acts per partner, condom use, needle sharing) differ by risk group: female sex workers, male clients of sex workers, men and women with non-regular partners, monogamous couples, MSM, and PWID	Behaviours (type of partners [regular, casual, or commercial sexual; injecting], sex acts per partner, condom use, needle sharing) differ by age and risk group (female sex workers, clients of sex workers, MSM, and PWID)	Number of short-term condomless sex partners in a 3-month period, and potentially one long-term condomless sex partner in a 3-month period	Sexual contact rates differ by risk group (low, medium, high) and by age; condoms are assumed to be used differentially by each risk group	Four types of sexual partnerships (marital, informal, transitory, and commercial) are remembered over time and formed according to specifiable partner age patterns
HIV acquisition determinants (including prevention interventions included)	Acquisition depends on characteristics of the individual (number of partners, circumcision status, PrEP use), the partner population (HIV prevalence, ART use, stage of infection), and the partnerships (sex acts per partner, prevalence of other STIs, type of sex, condom use)	Acquisition depends on characteristics (type of act [regular, casual, or commercial sexual; injecting], number of acts, circumcision and PMTCT status, and PrEP and PEP use), and population status (HIV testing, diagnosis, HIV prevalence, unsuppressive or suppressive ART use, stage of infection), in specified partnerships	Acquisition risk for each condomless sex partner depends on viral loads of people of (age-mixing relevant) opposite sex; circumcision and PrEP are modelled	Acquisition risk for each sex act depends on stage of HIV infection, ART status, circumcision status, PrEP use, and condom use	Acquisition risk depends on characteristics of an individual that include number of partners, circumcision status (males), condom use, other STIs, and coital acts; additionally, characteristics of the partner (including ART use if HIV positive and stage of infection) also determine an individual's acquisition risk
HIV natural history	Rate of decrease in CD4 cell count off of ART depends on current CD4 count and age; mortality off of ART depends on sex, age, and CD4 cell count; mortality on ART depends on CD4 cell count at initiation, age, sex, and duration on ART	Rate of decrease in CD4 cell count and viral load off of ART depends on current CD4 cell count and viral load; change in CD4 cell count on ART depends on current CD4 cell count and viral suppression of treatment; mortality both on and off of ART depends on current CD4 cell count and ART status (unsuppressive or suppressive)	Level and trend in viral load is dependent on sex and age; CD4 cell count decrease is dependent on viral load; risk of AIDS and death is dependent on current CD4 cell count, current viral load, and age	Rate of progression through each stage of infection is modelled on the basis of the mean duration of each stage; mortality on ART depends on whether ART was initiated at a CD4 count of ≥350 cells per μL or <350 cells per μL	HIV prognosis is calculated using a Weibull distribution where the parameters of the distribution are derived from CD4 cell count and age at the time of infection; CD4 cell count decreases from the time of infection; CD4 cell count increases if an individual initiates ART; new prognosis is calculated for ART dropouts
HIV testing and diagnosis	Testing is by type of test and population group and determines knowledge of status, but is not linked to transmission since ART coverage is a direct input	Testing is by type of test, population group, and year, which determines knowledge of status and allows linkage to care and initiation of ART on the basis of coverage level	A person without a diagnosis of HIV can be tested or not in each 3-month period; testing is indicated in antenatal clinics and for symptoms potentially of HIV, and general testing with various degrees of targeting at people with higher probability of infection	HIV testing and diagnosis is not explicitly represented but is considered a prerequisite for any initiation of treatment	HIV testing and diagnosis occurs voluntarily, at antenatal visits, or once symptomatic
ART	Risk of mortality while on ART is determined by age, sex, CD4 cell count at treatment initiation, and duration of treatment	Risk of mortality while on ART is determined by dynamically changing CD4 cell count over the course of treatment	Specific drugs and their current level of activity given drug resistance, and current ART adherence; being currently on ART has a small independent effect on risk of AIDS and HIV death over and above these factors; ART interruptions for reasons apart from disruptions are modelled; transmission of drug resistance	Risk of mortality while on ART is specific to whether ART was initiated early (CD4 count of ≥350 cells per μL) or late (CD4 count of <350 cells per μL)	Risk of mortality while on ART is determined by age, sex, CD4 cell count at treatment initiation, and duration of treatment
ART interruption	Immediate return to CD4 cell count at time of treatment initiation; survival progression is identical to those who are treatment naive	Substantial initial decrease in CD4 cell count towards pre-ART nadir (consistent with Grund et al^15^) and gradual return after interruption; viral load return is dependent on viral load monitoring	Immediate viral load return to pre-ART levels, substantial initial decrease in CD4 cell count towards pre-ART nadir (consistent with Grund et al)^15^	Mean survival time of 14 years after stopping ART, exponentially distributed, based on the survival time for HIV-positive individuals who have never been on ART (consistent with Todd et al^16^); a sensitivity analysis is also modelled using mean survival time of 32 years (Grund et al^15^) and 8 years after stopping ART (a hypothetical worst-case scenario); immediate return of death rate to pre-interruption levels on resumption of services with no effect on survival, except if individuals have already progressed to AIDS	CD4 cell count decreases after drop out from ART; prognosis is recalculated on the basis of age and CD4 cell count at the time of interruption of ART; calculation of ART prognosis after re-enrolling is identical to the initial enrolment with prognosis parameters corresponding to the age and CD4 cell count at re-enrolment
MTCT	Depends on the duration of breastfeeding and the CD4 cell count of the mother if no prophylaxis, or if on prophylaxis regimen, the type of regimen, retention at delivery, and interruption of ART during breastfeeding; retention on prophylaxis at delivery, duration of breastfeeding, and drop out from prophylaxis during breastfeeding	Depends on CD4 cell count of the mother, PMTCT coverage, and duration and breastfeeding practice	Dependent on viral load of the mother at birth	MTCT and PMTCT are not explicitly modelled	Depends on CD4 cell count of the mother if no prophylaxis, or prophylaxis regimen; retention on prophylaxis at delivery

Data are for adults and children, unless otherwise stated. ART=antiretroviral therapy. EMOD=Epidemiological MODeling software. MSM=men who have sex with men. MTCT=mother-to-child transmission. PEP=post-exposure prophylaxis. PMTCT=prevention of mother-to-child transmission. PrEP=pre-exposure prophylaxis. PWID=people who inject drugs. STI=sexually transmissible infection. VMMC=voluntary medical male circumcision.

Each model is based on many data sources; for instance, over 100 citations were used in the construction of the HIV Synthesis model. More details on the data used for each model are in the previous publications.^10^, ^11^, ^12^, ^13^, ^14^

### Analytical approach

All models simulate disruptions that last for 3 or 6 months starting from April 1, 2020, with 20%, 50%, or 100% of people who would otherwise benefit from a given service being affected, with our primary focus being on the 50% scenario. The potential disruptions to services are broadly grouped as prevention programmes, HIV testing, and treatment and care (table 2). At the end of the disruption period, service use is assumed to return to levels during the pre-disruption period. We considered the impacts on HIV mortality and incidence over 1-year and 5-year periods (table 2; appendix pp 4–5). We express results as the relative change in HIV deaths over the period of interest due to service disruption relative to the predicted annual number of such deaths in the relevant time period if no COVID-19 disruption or epidemic occurred. We used similar methods to describe the relative change in incidence of new HIV infections. We also considered the possibility that sexual activity will be decreased during the disruption period due to physical distancing measures (appendix pp 2–7). Using the results on the relative change in mortality due to interruption of ART, we calculated the predicted excess number of HIV deaths over 1 year in various example countries and sub-Saharan Africa as a whole.

**Table 2:** Predicted average relative change in HIV mortality and incidence over 1 year from April 1, 2020, due to a 6-month disruption of specific HIV services for 20%, 50%, and 100% of the population in countries in sub-Saharan Africa

		**Relative increase in HIV mortality**					**Relative increase in HIV incidence**				
		Goals	Optima HIV	HIV Synthesis	Imperial College London Model	EMOD	Goals	Optima HIV	HIV Synthesis	Imperial College London Model	EMOD
**Prevention programmes**											
Suspension of VMMC services											
	20% disruption	1·00 (1·00–1·00)	1·00 (1·00–1·00)	1·00 (0·99–1·01)	1·00 (1·00–1·00)	1·00 (1·00–1·10)*	1·00 (1·00–1·00)	1·00 (1·00–1·00)	1·00 (0·99–1·01)	1·00 (1·00–1·00)	1·00 (1·00–1·16)*
	50% disruption	1·00 (1·00–1·00)	1·00 (1·00–1·00)	1·00 (0·97–1·03)	1·00 (1·00–1·00)	1·00 (1·00–1·08)	1·00 (1·00–1·00)	1·00 (1·00–1·00)	1·00 (0·97–1·03)	1·00 (1·00–1·00)	1·00 (1·00–1·11)
	100% disruption	1·00 (1·00–1·00)	1·00 (1·00–1·00)	1·00 (0·95–1·07)	1·00 (1·00–1·00)	1·00 (1·00–1·07)*	1·01 (1·00–1·01)	1·00 (1·00–1·00)	1·00 (0·94–1·07)	1·00 (1·00–1·00)	1·00 (1·00–1·12)*
Condom availability interrupted											
	20% disruption	1·00 (1·00–1·00)	1·00 (1·00–1·00)	1·00 (0·99–1·01)	1·00 (1·00–1·00)	1·00 (1·00–1·07)*	1·07 (1·03–1·12)	1·02 (1·00–1·04)	1·01 (0·99–1·05)†	1·05 (1·05–1·05)	1·06 (1·00–1·20)
	50% disruption	1·00 (1·00–1·00)	1·00 (1·00–1·00)	1·00 (0·97–1·03)	1·00 (1·00–1·00)	1·00 (1·00–1·08)*	1·19 (1·07–1·30)	1·06 (1·01–1·10)	1·03 (0·99–1·13)†	1·12 (1·12–1·12)	1·14 (1·01–1·30)
	100% disruption	1·01 (1·00–1·01)	1·00 (1·00–1·00)	1·00 (0·95–1·06)	1·00 (1·00–1·00)	1·00 (1·00–1·07)	1·38 (1·15–1·62)	1·12 (1·02–1·20)	1·07 (0·98–1·28)†	1·25 (1·25–1·25)	1·28 (1·14–1·48)
Suspension of PMTCT											
	20% disruption	1·01 (1·00–1·02)	1·00 (1·00–1·01)	..	..	..	1·02 (1·00–1·05)	1·01 (1·00–1·02)	..	..	..
	50% disruption	1·03 (1·01–1·06)	1·01 (1·00–1·02)	..	..	..	1·05 (1·00–1·11)	1·02 (1·01–1·03)	..	..	..
	100% disruption	1·06 (1·02–1·11)	1·02 (1·01–1·04)	..	..	..	1·11 (1·00–1·23)	1·04 (1·01–1·07)	..	..	..
**HIV testing**											
Suspension of HIV testing											
	20% disruption	1·00 (1·00–1·02)	1·01 (1·00–1·02)	1·00 (0·99–1·02)†	..	1·00 (1·00–1·18)	1·00 (1·00–1·01)	1·00 (1·00–1·01)	1·00 (0·99–1·02)	..	1·00 (1·00–1·14)*
	50% disruption	1·01 (1·00–1·02)	1·01 (1·00–1·02)	1·01 (0·98–1·05)†	..	1·00 (1·00–1·18)*	1·01 (1·00–1·02)	1·01 (1·00–1·02)	1·01 (0·98–1·05)	..	1·01 (1·00–1·16)
	100% disruption	1·02 (1·00–1·02)	1·02 (1·00–1·03)	1·02 (0·96–1·11)†	..	1·00 (1·00–1·16)*	1·02 (1·00–1·04)	1·01 (1·00–1·02)	1·02 (0·96–1·10)	..	1·02 (1·00–1·18)
**Treatment and care**											
No new ART initiation											
	20% disruption	1·00 (1·00–1·02)	1·00 (1·00–1·00)	1·01 (0·99–1·02)†	1·01 (1·01–1·01)	1·00 (1·00–1·12)*	1·00 (1·00–1·01)	1·00 (1·00–1·00)	1·00 (0·99–1·02)	1·01 (1·01–1·01)	1·00 (1·00–1·15)
	50% disruption	1·01 (1·00–1·02)	1·00 (1·00–1·00)	1·02 (0·98–1·05)†	1·03 (1·03–1·03)	1·00 (1·00–1·13)*	1·01 (1·00–1·02)	1·00 (1·00–1·00)	1·00 (0·97–1·04)	1·02 (1·02–1·02)	1·02 (1·00–1·19)
	100% disruption	1·02 (1·00–1·02)	1·00 (1·00–1·00)	1·04 (0·98–1·14)†	1·06 (1·06–1·06)	1·00 (1·00–1·13)	1·02 (1·00–1·04)	1·00 (1·00–1·00)	1·01 (0·94–1·08)	1·04 (1·04–1·04)	1·03 (1·00–1·24)
Viral load testing, enhanced adherence counselling, and drug regimen switches stopped											
	20% disruption	1·01 (1·00–1·04)	1·01 (1·00–1·02)	1·00 (0·99–1·02)†	1·00 (1·00–1·00)	1·00 (1·00–1·08)	1·01 (1·00–1·04)	1·01 (1·00–1·02)	1·00 (0·99–1·02)	1·01 (1·01–1·01)	1·00 (1·00–1·11)*
	50% disruption	1·03 (1·00–1·10)	1·04 (1·03–1·05)	1·01 (0·98–1·05)†	1·00 (1·00–1·00)	1·01 (1·00–1·10)	1·03 (1·00–1·10)	1·02 (1·01–1·03)	1·00 (0·96–1·04)	1·03 (1·03–1·03)	1·00 (1·00–1·16)
	100% disruption	1·05 (1·00–1·20)	1·10 (1·07–1·11)	1·02 (0·97–1·11)†	1·00 (1·00–1·00)	1·03 (1·00–1·14)	1·05 (1·00–1·21)	1·05 (1·03–1·07)	1·00 (0·93–1·08)	1·07 (1·07–1·07)	1·00 (1·00–1·14)
Increase in death rate in people with AIDS-defining illnesses due to overstretched health system											
	20% disruption	1·01 (1·00–1·02)	..	1·02 (1·01–1·04)†	1·02 (1·02–1·02)	..	1·00 (1·00–1·00)	..	1·00 (0·99–1·01)	1·00 (1·00–1·00)	..
	50% disruption	1·02 (1·00–1·04)	..	1·06 (1·02–1·10)†	1·06 (1·06–1·06)	..	1·00 (1·00–1·00)	..	1·00 (0·97–1·03)	1·00 (1·00–1·00)	..
	100% disruption	1·05 (1·01–1·09)	..	1·12 (1·05–1·21)†	1·17 (1·16–1·17)	..	1·00 (1·00–1·00)	..	0·99 (0·94–1·07)	0·99 (0·99–0·99)	..
ART interruption											
	20% disruption	1·19 (1·11–1·28)	1·15 (1·11– 1·17)	1·29 (1·15–1·46)	1·25 (1·09–1·47)	1·34 (1·21–1·50)	1·06 (1·02–1·11)	1·04 (1·03–1·04)	1·02 (1·00–1·07)†	1·06 (1·06–1·07)	1·50 (1·29–1·69)
	50% disruption	1·55 (1·31–1·80)	1·39 (1·28–1·42)	1·87 (1·43–2·59)	1·63 (1·41–2·17)	1·83 (1·65–2·10)	1·07 (1·02–1·11)	1·09 (1·08–1·12)	1·06 (1·00–1·17)†	1·16 (1·15–1·18)	2·26 (1·97–2·51)
	100% disruption	2·18 (1·63–2·72)	1·75 (1·54–1·82)	3·51 (2·05–6·69)	2·27 (1·44– 3·35)	2·68 (2·40–3·10)	1·07 (1·02–1·12)	1·22 (1·18–1·25)	1·12 (1·01–1·38)†	1·32 (1·30–1·36)	3·49 (3·07–3·93)

Data are relative changes in estimates, with 95% uncertainty intervals in parentheses. For the Goals model, values are weighted averages of 13 countries in sub-Saharan Africa (South Africa, Malawi, Mozambique, Zimbabwe, eSwatini, Lesotho, Uganda, Kenya, Botswana, Tanzania, Cameroon, Côte d'Ivoire, and Nigeria). We assumed constant condom use rates and PMTCT coverage; historical rates of growth in VMMC; and adult and paediatric ART coverage increasing from 2019 levels to UNAIDS fast-track targets of 81% of all people who live with HIV on ART by 2025 for countries that are below those targets now or 90% if current coverage exceeds 81%.^10^ The VMMC, testing, and no new ART initiation disruptions affect the growth in the base case. For the increase in AIDS mortality due to overstretched health systems, we assumed that survival would be 2 years shorter with a complete failure of the health system and adjusted the age-specific, sex-specific, and CD4 cell count-specific survival rates accordingly to reflect the 6-month disruption affecting 20%, 50%, or 100% of the population. We assumed no change in sexual behaviour during the service disruption period. All estimates for this model are for adults and children, as relevant. For the Optima HIV model, all values are for all ages and are an average of 12 countries in sub-Saharan Africa (Botswana, Cameroon, Côte d'Ivoire, eSwatini, Kenya, Malawi, Mozambique, Nigeria, South Africa, Tanzania, Uganda, and Zimbabwe). Numbers of circumcisions are held constant over the disruption period because we assumed no new circumcisions would be done due to physical distancing concerns due to the COVID-19 pandemic. For the HIV Synthesis model, deaths and new HIV infections apply to adults only. 95% uncertainty intervals are the 2·5% and 97·5% percentiles of the distribution across setting scenarios and thus reflect uncertainty and intersetting variability. Suspension of PMTCT is not considered separately from interruption of all ART, which has an effect on MTCT. Estimates of disruption of PrEP programmes should be understood in the context that overall only 0·2% of women aged 15–25 years are on PrEP. An effect is seen on MTCT of interruption of ART, with an excess of 2·69 times more babies born with HIV in 1 year as a result of 6 months of disruption in 50% of people. For the Imperial College London model, figures in the table are an average of three countries in sub-Saharan Africa (Malawi, South Africa, and Zimbabwe) and are for adult mortality and new infections only. For survival estimates of individuals who have stopped ART (average monthly mortality risk of 0·24%, lower bound of average monthly mortality risk of 0·10%, and upper bound of 0·44%) more details are in the appendix (pp 17–18). Each scenario is modelled independently of other scenarios. For the EMOD model, to estimate the impact of condom availability interruption, transmission probability per sex act was increased during the disruption interval in proportion to the level of service disruption. Transmission risk factor returns to default values after the disruption period. ART=antiretroviral therapy. EMOD=Epidemiological MODeling software. MTCT=mother-to-child transmission. PMTCT=prevention of mother-to-child transmission. PrEP=pre-exposure prophylaxis. VMMC=voluntary medical male circumcision.

* Differences in estimates were non-significant given the stochastic variation.

† Data are significantly different from 1 —ie, no stochastic variability.

A summary of key aspects of each model is shown in table 1. Additional details on the approach we took for this analysis by each model are as follows. In the Goals model, coverage of all interventions except voluntary medical male circumcision (VMMC) were assumed to remain at 2019 levels, and VMMC coverage to increase according to current trends in recent years. Interruption of condom availability is modelled like all other behaviour change interventions, being suspended for the 3-month or 6-month periods, including community mobilisation, condom promotion, school-based programmes, and outreach services to key populations. The result is an increase in the number of sexual partners and a reduction in condom use. Modelling a reduction in sexual activity, as in the sensitivity analysis (appendix p 6), means that during the disruption period no casual or commercial sex occurs between heterosexual individuals or men who have sex with men (MSM). For suspension of prevention of mother-to-child transmission (PMTCT), the disruption means no mother-to-child transmission (MTCT) prophylaxis services are offered during the disruption period.

In the Optima HIV model, new VMMCs were assumed to stop over the disruption period. Change in sexual behaviour was represented by a 50% reduction in casual and commercial sexual contacts for applicable risk groups for the duration of the disruption, which return to pre-disruption levels immediately after the disruption period ends. Average values include results for 12 countries in sub-Saharan Africa (Botswana, Cameroon, Cote d'Ivoire, eSwatini, Kenya, Malawi, Mozambique, Nigeria, South Africa, Tanzania, Uganda, and Zimbabwe). Regional values are sums of country values located in the given region. The mortality rate for those on ART whose treatment is suspended was applied on the basis of data from Grund and colleagues.^15^ As a result, CD4 cell counts decrease rapidly and then gradually return to pre-ART levels after interruption ends. Once ART is resumed, the mortality rate for those on ART by CD4 cell count was applied. The impact on ART drug resistance as a result of disruption of HIV treatment and additional COVID-19-related deaths among people living with HIV were not included.

We used the HIV Synthesis model to generate a range of setting scenarios (n=300), with an aim to collectively represent the diversity of HIV epidemic and programme characteristics across and within countries in sub-Saharan Africa (appendix pp 11–12). For the effect of ART interruption, to generate country-specific estimates, we considered the association between the proportion of people with HIV who have viral suppression at the start of 2020 and the relative increase in death rate across setting scenarios. For this model, we present data for adult mortality only. Suspension of PMTCT services is not considered separately from interruption of all ART, which affects MTCT. We implemented the increase in the death rate among people with AIDS-defining illnesses due to overstretched health systems as a doubling in the death rate during the 3-month period in which an AIDS-defining illness occurs. Estimates of disruption of pre-exposure prophylaxis (PrEP) programmes are in the context of only 0·2% of women aged 15–25 years overall being on PrEP in the model. The assumptions in the model on changes in CD4 cell count during ART interruption result in a mortality rate of 1·7% in the first 3-month period of interruption due to disruption of ART availability and 5·0% during the second 3-month period. This model included effects of co-trimoxazole use for prevention of opportunistic infections, in addition to antiretroviral drugs. We also present the age breakdown of HIV deaths from this model.

To estimate the relative increase in death rate for a country, we referred to the proportion of people with HIV who have a suppressed viral load in the country to generate a country-specific estimate and applied the relative risk estimate to the annual number of adult HIV-related deaths from most recent UNAIDS 2018 estimates (appendix pp 13–15).^4^

The Imperial College London Model assumes that no new VMMCs occur during the disruption period, and that when the disruption ceases, circumcisions resume at the pre-disruption rate. For interruption of condom availability, condom use is assumed to be reduced by 50% for the duration of the disruption. ART initiation is assumed to be suspended such that no new individuals are initiated on ART for the duration of the disruption period and scale-up continues at pre-disruption rates after the disruption period ends, such that those who would have been initiated on ART during this period are allowed to start but might have progressed to a later stage of infection before starting. Viral load testing, enhanced adherence counselling, and switches are assumed to stop, and an additional 10% of individuals on ART are assumed to have an unsuppressed viral load. The death rate among people with AIDS-defining illnesses is assumed to increase due to overstretched health systems, such that the death rate for those with AIDS doubles for the duration of the disruption. A break in the supply of ART drugs, leading to ART interruption, is assumed to happen for 100% of individuals on ART, hence they stop using ART for the duration of the disruption period and are then re-initiated. If an individual has progressed to AIDS during this interruption, ART re-initiation is assumed to be too late for them to recover. Reduction in sexual contacts is assumed such that all sexual contacts across all risk groups decrease by 10% for the duration of the disruption period, and then return to pre-disruption levels immediately after the disruption period.

In the EMOD model, the scenario of VMMC interruption was assumed to apply only to in-clinic circumcisions provided by HIV prevention programmes; traditional male circumcision was assumed to continue at the pre-disruption rate. A disruption in condom availability was assumed to increase per-coital-act transmission probabilities, and after the disruption period transmission probabilities were assumed to return to their pre-disruption rates. A proportion of individuals who would have tested or initiated ART during the disruption period are unable to do so but might re-attempt testing and ART initiation after the disruption period ends at rates equivalent to pre-disruption rates among ART-naive individuals. Disruption of viral load testing, enhanced adherence counselling, and switches are assumed to increase the proportion of individuals with unsuppressed viral load during the disruption period.^18^ A break in supply of ART drugs leading to ART interruption is assumed to interrupt a proportion of ART use during the disruption period and resume ART use for those who survived the interruption to their ART or attempted to initiate ART during the disruption period. Survival during and after the disruption period depends on CD4 cell count and age, with older adults having more rapid decreases in CD4 cell count and increased risk of mortality. Viral load suppression after ART initiation, including re-initiation after a treatment interruption, is assumed to take an average of 3 months and up to 6 months. For scenarios with reduction in risky sexual behaviour during the service disruption period, we assumed a 50% reduction in risky sexual behaviour for commercial, informal, and transitory sexual contacts.

### Role of the funding source

The funder of the study had no role in study design, data collection, data analysis, data interpretation, or writing of the report. The corresponding author had full access to all the data in the study and had final responsibility for the decision to submit for publication.

## Results

Results for predicted effects of disruptions on various HIV services from the five models are shown in table 2 for 20%, 50%, and 100% of people being affected by the disruption, and predicted effects for the 50% scenario are shown in the figure. Disruptions to care and treatment services for people living with HIV are associated with increases in mortality risk, most notably disruption to the supply of ART drugs to people already on treatment resulting in discontinuation of ART. 6 months of such interruption to 50% of people is expected to lead to a 1·63 times (median over models; with the lowest estimate predicted using the Optima HIV model [1·39] and the highest by the HIV Synthesis model [1·87]) increase in HIV-related deaths over a 1-year period compared with no disruption. Although the effect in the first year is greatest, a substantial effect was also seen up to 5 years after the start of the disruption, such that a 6-month period of ART interruption for 50% of people is predicted to lead to an increase of 1·15–1·29 times the current annual number of deaths for each of the next 5 years (appendix pp 4–5). A 3-month interruption is predicted to lead to approximately half the excess HIV deaths estimated for the 6-month period (appendix pp 2–3). Although an interruption in the supply of ART drugs would have the largest impact of any potential disruptions, effects of poorer clinical care due to overstretched health facilities, interruptions of supply of other drugs such as co-trimoxazole, and suspension of HIV testing would all have a substantial effect on population-level mortality, with an up to a 1·06 times increase in HIV-related deaths over a 1-year period due to disruptions affecting 50% of the population compared with no disruption (table 2).

**Figure:**
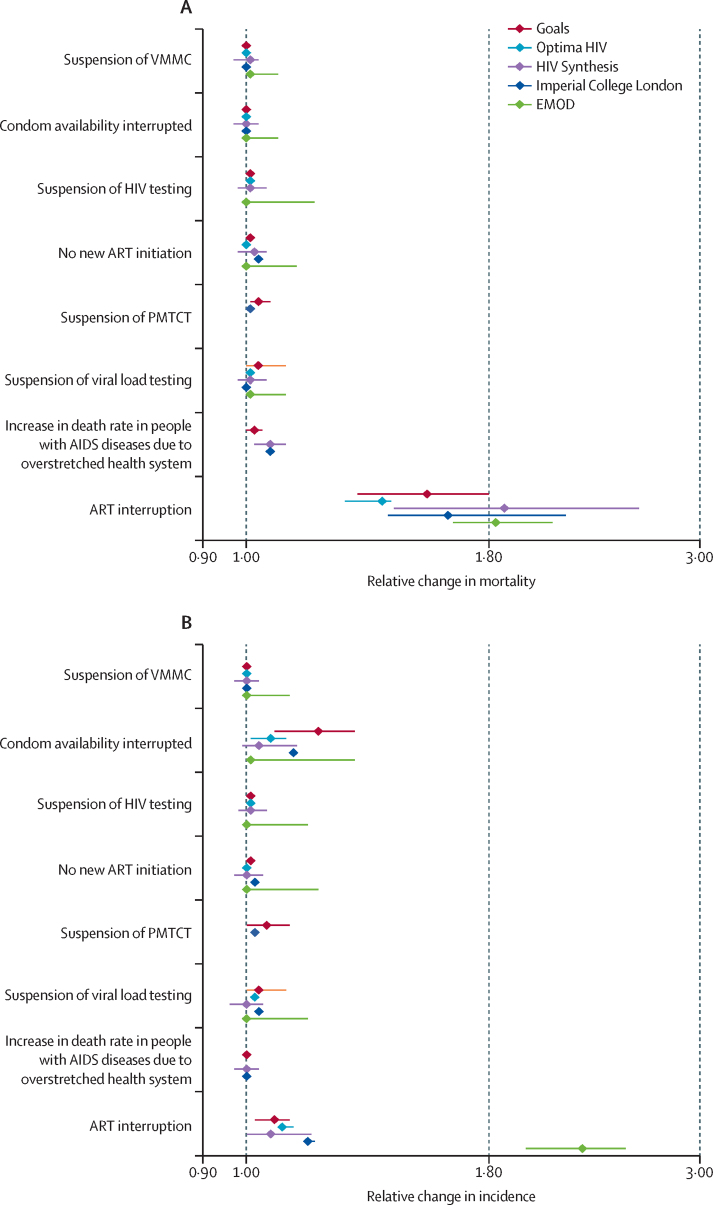
Predicted relative change in HIV mortality (A) and incidence (B) in 1 year from April 1, 2020, from a 6-month disruption of specific HIV services in sub-Saharan Africa, for 50% of the population Datapoints are point estimates with 95% uncertainty intervals indicated by whiskers. ART=antiretroviral therapy. EMOD=Epidemiological MODeling software. PMTCT=prevention of mother-to-child transmission. VMMC=voluntary medical male circumcision.

A disruption in VMMC programmes is predicted to have only a very small effect on HIV incidence (table 2). Disruption to outreach and condom programmes for 50% of people could lead to increases in the number of new infections of up to 1·19 times over 1 year. If reductions in sex with non-regular partners also occurred as a result of physical distancing, then the net effect could be a reduction in new infections (appendix pp 6–7). Longer-term effects over 5 years on HIV incidence are small (appendix pp 4–5). PrEP programmes are generally small in most settings, but a 6-month disruption in 50% of people is predicted to lead to a 1·005 times relative change in HIV incidence over 1 year (HIV Synthesis model; data not shown).

In table 3 we compare the predicted excess number of HIV deaths across models in countries and regions of sub-Saharan Africa resulting from a 6-month interruption of ART for 20%, 50%, and 100% of people on ART. For a 50% disruption, the median number of excess deaths in sub-Saharan Africa, across models, is estimated to be 296 000 (range 229 023–420 000). The proportion of such HIV deaths that occur in people younger than 65 years is estimated to be 95% (HIV Synthesis model; data not shown).

**Table 3:** Predicted excess HIV-related deaths over 1 year from April 1, 2020, due to a 6-month interruption of ART for 20%, 50%, and 100% of the population in countries in sub-Saharan Africa

		Estimated HIV-related deaths in 2018*	**Excess HIV-related deaths over 1 year: 2020–21**				
			Goals	Optima HIV	HIV Synthesis	Imperial College London Model†	EMOD
South Africa		71 000 (52 000–91 000)	..	..	..	..	..
	20% disruption	..	35 000 (27 000–34 000)	19 000 (15 000–24 000)	14 500 (10 600–20 500)	16 000 (5000–32 000)	27 614 (26 000–29 228)
	50% disruption	..	100 000 (80 000–130 000)	45 000 (36 000–57 000)	42 200 (30 400–64 000)	40 100 (12 500–80 000)	68 519 (66 600–70 500)
	100% disruption	..	230 000 (170 000–280 000)	84 000 (66 000–107 000)	112 000 (74 600–190 000)	80 400 (25 000–160 300)	138 126 (135 400–140 900)
Malawi		13 000 (11 000–16 000)	..	..	..	..	..
	20% disruption	..	4800 (3800–5700)	3800 (3700–4000)	3200 (1800–4200)	2600 (900–5200)	4582 (3900–5500)‡
	50% disruption	..	14 000 (11 000–17 000)	9300 (8900–9900)	9900 (6100–14 000)	6600 (2200–12 900)	11 370 (9600–13 600)‡
	100% disruption	..	32 000 (25 000–38 000)	18 000 (17 000–19 000)	29 200 (12 900–46 700)	13 200 (4300–25 900)	22 921 (19 400–27 400)‡
Mozambique		54 000 (39 000–73 000)	..	..	..	..	..
	20% disruption	..	11 000 (6500–16 000)	12 000 (9700–16 000)	9500 (5900–14 000)	13 500 (4900–25 400)‡	..
	50% disruption	..	32 000 (19 000–46 000)	29 000 (23 000–37 000)	27 500 (16 700–34 200)	34 000 (22 100–63 200)‡	..
	100% disruption	..	69 000 (40 000–98 000)	54 000 (44 000–69 000)	72 000 (39 200–94 000)	68 600 (23 800–126 900)‡	..
Zimbabwe		22 000 (17 000–27 000)	..	..	..	..	..
	20% disruption	..	3500 (2500–4400)	2700 (2500–3000)	5000 (2800–6900)	4200 (1200–8300)	5146 (4200–6200)‡
	50% disruption	..	12 000 (8600–15 000)	8500 (7900–9500)	15 100 (7500–22 100)	10 500 (3400–20 800)	12 768 (10 400–15 300)‡
	100% disruption	..	29 000 (21 000–37 000)	21 000 (18 000–24 000)	42 300 (18 100–70 300)	21 100 (6800–41 600)	25 738 (21 000–30 800)‡
eSwatini		2400 (2000–2900)	..	..	..	..	..
	20% disruption	..	550 (430–670)	1100 (900–1200)	770 (470–970)	600 (200–1100)‡	1130 (1000–1300)‡
	50% disruption	..	1600 (1300–2000)	2500 (2200–3200)	2570 (1670–3470)	1500 (1000–2800)‡	2803 (2400–3200)‡
	100% disruption	..	3600 (2800–4400)	4700 (2600–7000)	8270 (4670–12 670)	3000 (1100–5600)‡	5651 (4800–6500)‡
Lesotho		6100 (5000–7500)	..	..	..	..	..
	20% disruption	..	2500 (2000–2900)	..	1200 (600–1700)	1500 (500–2800)‡	1287 (1100–1400)‡
	50% disruption	..	6700 (5500–7900)	..	4400 (1600–5200)	3800 (2500–7000)‡	3194 (2800–3600)‡
	100% disruption	..	13 000 (11 000–16 000)	..	9900 (3700–15 400)	7600 (2600–14 100)‡	6438 (5600–7200)‡
Uganda		23 000 (19 000–31 000)	..	..	..	..	..
	20% disruption	..	10 000 (7400–13 000)	9600 (8200–12 000)	2300 (2300–6500)	5800 (2100–10 800)‡	5950 (5200–6900)‡
	50% disruption	..	26 000 (19 000–33 000)	23 000 (19 000–28 000)	15 300 (6300–20 500)	14 500 (9400–26 900)‡	14 765 (12 900–17 100)‡
	100% disruption	..	52 000 (37 000–66 000)	43 000 (36 000–53 000)	43 700 (14 800–64 600)	29 200 (10 100–54 100)‡	29 765 (25 900–34 400)‡
Kenya		25 000 (18 000–38 000)	..	..	..	..	..
	20% disruption	..	12 000 (6200–18 000)	8300 (5900–12 000)	6000 (3400–8040)	6300 (2300–11 800)‡	7608 (5900–9400)‡
	50% disruption	..	32 000 (16 000–48 000)	20 000 (14 000–29 000)	18 900 (9800–26 700)	15 800 (10 300–29 300)‡	18 878 (14 700–23 300)‡
	100% disruption	..	66 000 (34 000–99 000)	38 000 (26 000–55 000)	55 900 (24 700–89 400)	31 800 (11 000–58 800)‡	38 056 (29 700–47 000)‡
Botswana		4800 (4100–5700)	..	..	..	..	..
	20% disruption	..	840 (650–1000)	1800 (1500–2400)	1700 (1200–2100)	1200 (400–2300)‡	1990 (1600–2300)‡
	50% disruption	..	2700 (2100–3300)	4700 (3900–6200)	5400 (3500–7400)	3000 (2000–5600)‡	4939 (4000–5800)‡
	100% disruption	..	6100 (4700–7600)	9500 (7900–12 000)	17 400 (9800–26 800)	6100 (2100–11 300)‡	9956 (8000–11 700)‡
Tanzania		24 000 (20 000–29 000)	..	..	..	..	..
	20% disruption	..	8900 (7100–11 000)	5300 (3600–7500)	5200 (2400–6700)	6300 (2300–11 800)‡	6588 (5200–7700)‡
	50% disruption	..	26 000 (20 000–31 000)	13 000 (9000–18 000)	15 800 (6500–21 200)	15 800 (10 300–29 300)‡	16 347 (12 900–19 100)‡
	100% disruption	..	54 000 (44 000–65 000)	24 000 (16 000–35 000)	45 200 (15 300–66 800)	31 800 (11 000–58 800)‡	32 954 (26 000–38 400)‡
Cameroon		18 000 (15 000–21 000)	..	..	..	..	..
	20% disruption	..	3600 (2800–4400)	3400 (2700–4500)	3300 (1900–4400)	4300 (1500–8000)‡	2847 (2400–3200)‡
	50% disruption	..	10 000 (8000–13 000)	8200 (6300–11 000)	8700 (5300–10 900)	10 100 (7000–19 900)‡	7065 (6000–7900)‡
	100% disruption	..	23 000 (18 000–29 000)	15 000 (12 000–20 000)	22 900 (12 400–29 900)	21 600 (7500–40 000)‡	14 243 (12 100–15 900)‡
Côte d'Ivoire		16 000 (11 000–23 000)	..	..	..	..	..
	20% disruption	..	1900 (1100–2800)	2700 (2100–3600)	2900 (1800–4200)	4000 (1400–7500)‡	1253 (800–2000)‡
	50% disruption	..	5600 (3100–8200)	6600 (5000–8800)	8400 (5100–10 400)	10 100 (6600–18 700)‡	3108 (2000–5000)‡
	100% disruption	..	12 000 (6500–17 000)	12 000 (9200–16 000)	21 900 (11 900–28 600)	20 300 (7000–37 600)‡	6265 (3900–10 000)‡
Nigeria		53 000 (31 000–89 000)	..	..	..	..	..
	20% disruption	..	8800 (1800–16 000)	12 000 (9000–16 000)	8400 (5200–12 400)	13 300 (4800–25 000)‡	5300 (3000–9800)‡
	50% disruption	..	25 000 (5000–44 000)	28 000 (21 000–37 000)	24 400 (14 800–30 400)	33 400 (21 700–62 000)‡	13 150 (7400–24 400)‡
	100% disruption	..	50 000 (10 000–90 000)	52 000 (39 000–69 000)	64 000 (34 800–83 600)	67 300 (23 300–124 600)‡	26 509 (14 900–49 200)‡
Eastern and southern Africa		310 000 (230 000–400 000)	..	..	..	..	..
	20% disruption	..	110 000 (73 000–150 000)	77 000 (68 000–106 000)	83 700 (46 500–114 700)	77 500 (27 900–145 700)‡	79 349 (60 400–101 700)‡
	50% disruption	..	320 000 (210 000–440 000)	186 000 (149 000–237 000)	251 100 (124 000–365 800)	195 300 (127 100–362 700)‡	196 889 (150 000–252 300)‡
	100% disruption	..	690 000 (450 000–930 000)	351 000 (279 000–450 000)	700 600 (300 700–1 165 600)	393 700 (136 400–728 500)‡	396 905 (302 300–508 700)‡
Western and central Africa		160 000 (110 000–230 000)	..	..	..	..	..
	20% disruption	**..**	34 000 (17 000–50 000)	31 000 (24 000–41 000)	33 600 (20 800–49 600)	40 000 (14 400–75 200)‡	12 950 (6600–22 200)‡
	50% disruption	..	96 000 (49 000–140 000)	73 000 (56 000–97 000)	97 600 (59 200–121 600)	100 800 (65 600–187 200)‡	32 134 (16 500–55 000)‡
	100% disruption	**..**	200 000 (100 000–300 000)	138 000 (104 000–183 000)	256 000 (139 200–334 400)	203 200 (70 400–376 000)‡	64 778 (33 200–110 900)‡
Sub-Saharan Africa		470 000 (340 000–630 000)	..	..	..	..	..
	20% disruption	**..**	150 000 (90 000–200 000)	107 000 (92 000–147 000)	117 300 (67 300–164 300)	117 500 (42 300–220 900)‡	92 299 (67 100–123 900)‡
	50% disruption	**..**	420 000 (260 000–580 000)	259 000 (206 000–335 000)	348 700 (163 200–487 400)	296 100 (192 700–549 900)‡	229 023 (166 400–307 300)‡
	100% disruption	**..**	890 000 (540 000–1 200 000)	488 000 (383 000–633 000)	956 600 (439 900–1 500 000)	596 900 (206 800–1 104 500)‡	461 682 (335 500–619 600)‡

Data are estimates with 95% uncertainty intervals. Data are for adults and children, unless otherwise stated, and analyses assume no change in sexual behaviour associated with the period of disruption. In the EMOD model, the number of deaths during an ART interruption in each setting is assumed to be proportional to the number of individuals on ART with viral load suppression at the time of the interruption. In the HIV Synthesis model, values are for adults only (aged ≥15 years). ART=antiretroviral therapy. EMOD=Epidemiological MODeling software.

* UNAIDS estimates for 2019.^4^

† Numbers in parentheses are 95% uncertainty intervals for survival estimates of individuals who have stopped ART as described in table 1.

‡ Estimated by applying the relative increase in HIV mortality over 1 year from table 2 to estimated HIV-related deaths in 2018 by country.

A suspension of PMTCT activities for 3 months could lead to large increases in the number of new child infections according to the Optima HIV and Goals models. For example, in the Goals model, suspension of these activities leads to relative increases in child infections of 1·81 times in Malawi, 1·41 times in Mozambique, 1·70 times in Uganda, and 1·53 times in Zimbabwe because PMTCT coverage is quite high in most countries. The HIV Synthesis model identified the impact on MTCT of interruption of ART, with 1·64 times more babies born with HIV in 1 year as a result of a 6-month disruption in 50% of people.

## Discussion

A 6-month interruption in ART supplies for 50% of people would be expected to lead to an approximately 1·63 times (range 1·39–1·87) increase in HIV-related deaths over 1 year. In sub-Saharan Africa, this increase amounts to a median of 296 000 (range 229 023–420 000) excess HIV deaths over this period. This substantial number of excess deaths can be explained by the fact that CD4 lymphocyte cell count recovery, which takes years to achieve on ART, is rapidly lost after viral replication resumes in the absence of ART.

Although an interruption in ART drug supply would have by far the largest impact of any potential disruption, effects of lower quality clinical care due to overstretched health facilities, interruption of supply of other drugs (eg, co-trimoxazole, which is taken to prevent protozoal, bacterial, and fungal infections), and suspension of HIV testing, or due to reluctance among patients to access care because of concerns about exposure to severe acute respiratory syndrome coronavirus 2^19^ would all have substantial population-level effects. Interruption to condom supplies, PrEP, and peer education would make populations more susceptible to increases in HIV incidence, although physical distancing measures could lead to reductions in risky sexual behaviour.

We chose to study the effects of disruptions to 50% of affected populations. We aimed to show the areas that are most susceptible to disruption among the various services that form part of most countries' national HIV responses and our results should not be taken as a prediction that disruption will be as extensive as estimated here. Such an extensive disruption to ART access as estimated here seems unlikely unless, for example, a country's supply of ART drugs is delayed in being dispatched from the factory or in transit. On July 6, 2020, WHO announced that 73 countries have warned that they are at risk of stock-outs of antiretroviral medicines as a result of the COVID-19 pandemic.^20^ As of July 29, 2020, The President's Emergency Plan for AIDS Relief reported that the median delay in reciving ART drugs is 35 days for adults and 29 days for children.^21^ Deliveries of orders for antiretrovirals are delayed because most manufacturers are based in India (which has been under lockdown in response to COVID-19) and transport challenges. Incoming raw materials from China to India have also been reported to be affected by logistical constraints. Anecdotal evidence from Malawi shows relaxation of eligibility criteria for multi-month dispensing of ART drugs, with around 30–40% of people who live with HIV on ART being on a 6-month refill compared with 10% during the pre-COVID-19 era (Chagoma N, unpublished). We did not specifically consider disruptions of services to key populations, such as female sex workers or homosexual men and other MSM but, given the levels of stigma around these populations, they could be particularly susceptible to interruptions in services. Condom access and other prevention services outside health facilities might be particularly affected at times when movement is restricted. Some form of disruptions affecting sections of the population could last longer than 6 months, especially if health systems are severely affected by loss of personnel or fear of returning to health-care settings if personal protective equipment is not available or access is restricted, and monitoring such effects will be important.

While some uncertainty exists around the magnitude of the effect that programme disruptions would have on the HIV epidemic, which is reflected in the range of predictions across the models, interruption of ART is generally agreed to be the greatest threat to HIV mortality and incidence. The differences across models in predicted consequences of ART interruption result largely from differences in how interruptions are modelled, and the consequent death rate assumed. Two of the models (Optima HIV and HIV Synthesis) directly consider the CD4 cell count decrease after interruption. The Goals model assumes an immediate return to the pre-ART death rate after interruption, and the Imperial College London model calculates the death rate on the basis of the mean time to progress to AIDS in the absence of ART. Although data are available on changes in CD4 cell count after interruption,^15^ little direct data exist on death rates because no large scale studies of effects of complete interruption of ART for substantial periods have been done in which people have been observed longitudinally after interruption of ART because of the known harmful effects of stopping ART. All the models predict that ART interruption, in the absence of changes in sexual behaviour, will lead to increases in HIV incidence. The EMOD model predicts that this increase would be particularly large, perhaps due to transmission during acute HIV infection causing a synchronous wave of secondary spread, which might be more pronounced in a network model.

Although the largest effect of disruptions to HIV programmes would occur as a result of ART interruption, effects on prevention, testing, and other aspects of treatment and care could be clinically significant. These programmes are generally highly effective and cost-effective, so any loss to their functioning will lead to adverse health consequences for many people. Some short-term changes to HIV programmes could have further long-term ramifications not represented in the 5-year timeline. For example, reductions in peer support might result in suboptimal treatment adherence and retention in care. Health-care provisions of all types, for HIV and other diseases and conditions, should be prioritised for maintenance, with modifications if needed, in so far as can be safely attained during the period of threat from COVID-19. Multi-month dispensing for PrEP and ART users and extra emergency provision of supplies are probably required to safely provide existing levels of health care at the same time as responding to COVID-19.

Previous assessments have considered effects on HIV care in other crisis situations such as the post-election unrest in Kenya in 2007,^22^, ^23^ during the Ebola epidemic in Guinea in 2014,^24^ in acute conflict areas in the Central African Republic and Yemen,^25^ and as a consequence of food insecurity.^26^ All assessments found substantial negative effects but also evidence of resilience and ability to successfully ensure access to ART drugs.^25^, ^26^ By contrast with concern over negative effects of the COVID-19 pandemic, longer-term benefits might be seen because the crisis has led to simplifications to delivery of care, with multi-month dispensing and fewer clinic visits required per patient than previously, which in turn reduces costs and results in a similar health outcome.^27^

Our model predictions are inherently limited by the available data to inform them. Although data on HIV and effects of programmes implementing interventions are available, some key uncertainties exist. One of these uncertainties is the magnitude of the effect of interruption of ART on death rates. Other uncertainties that might also be important include that we did not model any interaction between HIV and COVID-19—eg, that people with HIV were not assumed to be more or less likely to acquire or die from COVID-19, although any excess COVID-19 risk is likely to be small in absolute terms compared with risk of death directly due to HIV. We also note that several models address excess mortality due to disruption of services for HIV and tuberculosis, malaria, and other communicable diseases^3^, ^28^, ^29^, ^30^ and all of these models point to similar levels of excess death and we recognise that overlaps can exist in people counted as having died separately of tuberculosis and HIV. However, taken together, our models highlight the real risk of substantially increased mortality among the most susceptible and at-risk populations in sub-Saharan Africa and could quickly erase years of public health gains if action to mitigate these effects are not swift and coordinated.

In summary, during the COVID-19 pandemic the primary priorities for governments, donors, suppliers, and communities to avoid additional HIV-related deaths should focus on maintaining an uninterrupted supply of ART drugs for people with HIV. The provision of other HIV prevention measures is also important to prevent any increase in HIV incidence.
