# Supplementary material for: Potential effects of disruption to HIV programmes in sub-Saharan Africa caused by COVID-19: results from multiple mathematical models
Source: Lancet HIV. 2020 Aug 6;7(9):e629–40. doi: 10.1016/S2352-3018(20)30211-3 (PMC7482434; doi:10.1016/S2352-3018(20)30211-3)
Supplement: Supplementary appendix [file mmc1.pdf]

THE LANCET
HIV

Supplementary appendix

This appendix formed part of the original submission and has been peer reviewed. We post it as supplied by the authors.

Supplement to: Jewell BL, Mudimu E, Stover J, et al. Potential effects of disruption to HIV programmes in sub-Saharan Africa caused by COVID-19: results from multiple mathematical models. *Lancet HIV* 2020; published online Aug 6. http://dx.doi.org/10.1016/S2352-3018(20)30211-3.

Potential effects of disruption to HIV programmes in sub-Saharan Africa caused by COVID-19: results from multiple mathematical models

APPENDIX

Further results and modelling details

Table A1. Predicted average relative increase in HIV mortality and incidence in 1 year from 1 April 2020 in countries in sub-Saharan Africa that would result from a 3-month disruption of specific HIV services for 50% of the population, with 95% uncertainty bounds. Assuming no change in sexual behaviour associated with the period of disruption. For adults and children, except where stated.

HIV service disruption	Relative increase in HIV mortality in 1 year over 2020-2021 from a 3-month disruption of services					Relative increase in HIV incidence in 1 year over 2020-2021 from a 3-month disruption of services				
	Goals	Optima HIV	HIV Synthesis	Imperial Model	EMOD	Goals	Optima HIV	HIV Synthesis	Imperial Model	EMOD
Suspension of VMMC services	1.00 (1.00 – 1.00)	1.00 (1.00 – 1.00)	1.00 (0.94 – 1.04)	1.00 (1.00 – 1.00)	1.00*(1.00 – 1.07)	1.00 (1.00 – 1.00)	1.00 (1.00 – 1.00)	1.00 (0.95 – 1.06)	1.00 (1.00 – 1.00)	1.01 (1.00 – 1.16)
Condom availability interrupted	1.00 (1.00 – 1.00)	1.00 (1.00 – 1.00)	1.00 (0.95 – 1.05)	1.00 (1.00 – 1.00)	1.00 (1.00 – 1.08)	1.09 (1.03 – 1.15)	1.02 (1.01 – 1.03)	1.02(0.95 – 1.07)+	1.06 (1.06 – 1.06)	1.07 (1.00 – 1.22)
Suspension of HIV testing	1.01 (1.00 – 1.01)	1.01 (1.00 – 1.02)	1.01(0.96 – 1.06)+	---	1.00*(1.00 – 1.17)	1.01 (1.00 – 1.01)	1.00 (1.00 – 1.00)	1.00(0.98 – 1.05)+	---	1.01 (1.00 – 1.18)
No new ART initiation	1.01 (1.00 – 1.01)	1.00 (1.00 – 1.00)	1.01(0.97 – 1.05)+	1.02 (1.01 – 1.02)	1.00*(1.00 -1.14)	1.01 (1.00 – 1.01)	1.00 (1.00 – 1.00)	1.00 (0.95 – 1.05)	1.01 (1.01 – 1.01)	1.02 (1.00 – 1.16)
Suspension of PMTCT	1.02 (1.00 – 1.03)	1.01 (1.00 – 1.01)	---	---	---	1.03 (1.00 – 1.06)	1.01 (1.00 – 1.01)	---	---	---
Viral load testing, enhanced adherence counselling and drug regimen switches stopped	1.01 (1.00 – 1.05)	1.02 (1.01 – 1.03)	1.01(0.95 – 1.06)+	1.00 (1.00 – 1.00)	1.00 (1.00 – 1.09)	1.01 (1.00 – 1.05)	1.01 (1.00 – 1.02)	1.00 (0.95 – 1.05)	1.02 (1.02 – 1.02)	1.03 (1.00 – 1.15)

Increase in death rate in people with AIDS diseases due to over-stretched health system	1.00 (1.00 – 1.00)	---	1.03(0.98 –1.07)+	1.03 (1.03 – 1.03)	---	1.01 (1.00 – 1.02)	---	1.00(0.96 – 1.07)+	1.00 (1.00 – 1.00)	---
ART interruption	1.25 (1.14 – 1.37)	1.17 (1.11 – 1.23)	1.53(1.25– 1.67)+	1.36 (1.12 – 1.66)	1.62(1.46 – 1.89)	1.06 (1.01 – 1.11)	1.06 (1.04 – 1.07)	1.04(0.96 – 1.12)+	1.09 (1.08 – 1.10)	1.89 (1.63 – 2.10)

See footnotes to Table 2 in main paper.

Table A2. Predicted average relative annual increase in HIV mortality and incidence in 5 years from 1 April 2020 in countries in sub-Saharan Africa that would result from a 6-month disruption of specific HIV services for 50% of the population, with 95% uncertainty bounds. Assuming no change in sexual behaviour associated with the period of disruption. For adults and children, except where stated.

HIV service disruption	Relative increase in HIV mortality in 5 years over 2020-2024 from a 6-month disruption of services					Relative increase in HIV incidence in 5 years over 2020-2024 from a 6-month disruption of services				
	Goals	Optima HIV	HIV Synthesis*	Imperial Model	EMOD	Goals	Optima HIV	HIV Synthesis	Imperial Model	EMOD
Suspension of VMMC services	1.00 (1.00 – 1.00)	1.00 (1.00 – 1.00)	1.00 (0.95 – 1.04)	1.00 (1.00 – 1.00)	1.00 (1.00 – 1.05)	1.00 (1.00 – 1.00)	1.00 (1.00 – 1.00)	1.00 (0.93 – 1.07)	1.00 (1.00 – 1.00)	1.01 (1.00 – 1.07)
Condom availability interrupted	1.00 (1.00 – 1.01)	1.01 (1.00 – 1.02)	1.00 (0.96 – 1.04)	1.00 (1.00 – 1.00)	1.00 (1.00 – 1.05)	1.03 (1.01 – 1.06)	1.02 (1.01 – 1.03)	1.01 (0.96 -1.10)+	1.02 (1.02 – 1.02)	1.03 (1.00 – 1.11)
Suspension of HIV testing	1.00 (1.00 – 1.01)	1.01 (1.00 – 1.02)	1.01 (0.97 -1.05)+	---	1.00 (1.00 – 1.08)	1.00 (1.00 – 1.01)	1.01 (1.00 – 1.02)	1.00 (0.94 – 1.08)	---	1.01 (1.00 – 1.09)
No new ART initiation	1.00 (1.00 – 1.01)	1.00 (1.00 – 1.00)	1.01 (0.98 -1.04)+	1.01 (1.01 – 1.01)	1.01 (1.00 – 1.07)	1.00 (1.00 – 1.01)	1.00 (1.00 – 1.00)	1.00 (0.95 – 1.10)	1.02 (1.02 – 1.02)	1.01 (1.00 – 1.08)
Suspension of PMTCT	1.01 (1.00 – 1.02)	1.01 (1.00 – 1.01)	---	---	---	1.01 (1.00 – 1.02)	1.01 (1.00 – 1.02)	---	---	---
Viral load testing, enhanced adherence counselling and drug regimen switches stopped	1.00 (1.00 – 1.02)	1.01 (1.00 – 1.02)	1.01 (0.97 -1.05)+	1.00 (1.00 – 1.00)	1.01 (1.00 – 1.06)	1.00 (1.00 – 1.02)	1.01 (1.00 – 1.02)	1.00 (0.94 – 1.06)	1.01 (1.01 – 1.01)	1.02 (1.00 – 1.09)
Increase in death rate in people with AIDS diseases	1.00 (1.00 – 1.00)	---	1.00 (0.95 – 1.04)	1.00 (1.00 – 1.00)	---	1.00 (1.00 – 1.01)	---	1.00 (0.93 – 1.07)	1.00 (1.00 – 1.00)	---

due to over-stretched health system										
ART interruption	1.22 (1.01 – 1.34)	1.15 (1.09 – 1.21)	1.29(1.11 -1.50)+	1.22 (1.17 – 1.39)	1.24 (1.16 – 1.31)	1.03 (1.01 – 1.04)	1.05 (1.04 – 1.06)	1.01 (0.90-1.25)+	1.04 (1.04 – 1.05)	1.25 (1.16 – 1.34)

See footnotes to Table 2 in main paper.

Table A3. Predicted average relative increase in HIV mortality and incidence in 1 year from 1 April 2020 over 2020 in countries in sub-Saharan Africa that would result from a 6-month disruption of specific HIV services for 50% of the population, with 95% uncertainty bounds. Assuming a reduction in sexual risk behaviour associated with the period of disruption. For adults and children, except where stated.

HIV service disruption	Relative increase in HIV mortality in 1 year over 2020-2021 from a 6-month disruption of services					Relative increase in HIV incidence in 1 year over 2020-2021 from a 6-month disruption of services				
	Goals	Optima HIV	HIV Synthesis*	Imperial Model	EMOD	Goals	Optima HIV	HIV Synthesis	Imperial Model	EMOD
Suspension of VMMC services	1.00 (1.00 – 1.00)	1.00 (1.00 – 1.00)	1.00 (0.95 - 1.05)	1.00 (1.00 – 1.00)	0.99 (0.91 – 1.09)	0.84 (0.78 – 0.91)	0.90 (0.88 – 0.92)	0.95(0.91 - 1.04)+	0.89 (0.89 – 0.89)	0.90 (0.79 – 1.03)
Condom availability interrupted	1.00 (1.00 – 1.00)	1.00 (1.00 – 1.00)	1.00 (0.96 - 1.04)	1.00 (1.00 – 1.00)	0.99 (0.93 – 1.07)	0.91 (0.85 –0.96)	0.95 (0.93 – 0.96)	0.96 (0.90 - 1.03)+	0.94 (0.94 – 0.94)	1.01 (0.89 – 1.16)
Suspension of HIV testing	1.00 (1.00 – 1.01)	1.01 (1.01 – 1.02)	1.02 (0.97 -1.08) +	---	0.99 (0.82 – 1.16)	0.84 (0.78 – 0.91)	0.99 (0.99 – 0.99)	0.95 (0.87 - 1.02)+	---	0.90 (0.78 – 1.06)
No new ART initiation	1.00 (1.00 – 1.01)	1.00 (1.00 – 1.00)	1.02 (0.97 - 1.07)+	1.03 (1.03 – 1.03)	0.99 (0.91 – 1.12)	0.84 (0.78 – 0.91)	0.90 (0.88 – 0.92)	0.95 (0.87 - 1.02)+	0.87 (0.87 – 0.87)	0.93 (0.79 – 1.07)
Suspension of PMTCT	1.01 (1.00 – 1.03)	1.00 (1.00 – 1.00)	---	---	---	0.86 (0.79 –0.94)	0.97 (0.96 – 0.98)	---	---	---
Viral load testing, enhanced adherence counselling and drug	1.02 (0.97 – 1.05)	1.04 (1.04 - 1.05)	1.01 (0.96 - 1.07)+	1.00 (1.00 – 1.00)	1.01 (0.95 – 1.10)	0.84 (0.75 – 0.93)	0.90 (0.87 – 0.93)	0.95 (0.86 - 1.02) +	0.88 (0.88 – 0.88)	0.90 (0.81 – 1.05)

regimen switches stopped										
Increase in death rate in people with AIDS diseases due to over-stretched health system	1.01 (1.00 – 1.02)	---	1.06(1.01 - 1.13) +	1.06 (1.06 – 1.06)	---	0.84 (0.77 –0.90)	---	0.95 (0.87 - 1.01)+	0.85 (0.85 – 0.85)	---
ART interruption	1.49 (1.26 – 1.73)	1.32 (1.22 – 1.35)	1.94 (1.50 - 2.66)+	1.63 (1.22 – 2.17)	1.83 (1.64 -2.10)	0.89 (0.82 – 0.97)	0.94 (0.93 – 0.95)	0.99 (0.90 - 1.09)+	1.00 (0.99 – 1.02)	2.08 (1.79 – 2.31)

See footnotes to Table 2 in main paper.

Optima HIV model

Estimation of the potential effects of disruption to HIV programs in sub-Saharan Africa caused by COVID-19: additional information for the Optima HIV model

Additional information

1. Methods

Existing national-level Optima HIV models for 12 countries in sub-Saharan Africa including for Botswana, Cameroon, Cote d'Ivoire, Eswatini, Kenya, Malawi, Mozambique, Nigeria, South Africa, Tanzania, Uganda, and Zimbabwe were used. An Optima HIV model for Lesotho is not available. Individual national estimates were generated, and respective values aggregated for the two subregions (Eastern and Southern Africa and Western and Central Africa) and for sub-Saharan Africa, with estimates extrapolated to represent regional burden of people living with HIV for each region.

The impact on drug resistance as a result of this disruption and additional COVID-19-related deaths among people living with HIV (PLHIV) were not considered.

The HIV-related mortality rate was applied to those on antiretroviral therapy (ART) who were removed from ART due to this disruption following empirical observations from the SMART Study Group (1). CD4 counts declined rapidly as a result, and gradually returned to pre-ART levels after interruption. Once ART coverage was resumed following the disruption, mortality rate for those on ART were reapplied.

2. CD4 progression

Times for CD4 progression used in the Optima HIV model are listed in Table A4.

Table A4. Average times in years to progress to the next CD4 stage

Stage	Average time to progress to next CD4 stage in years (low-high)
Acute to >500	0.24 (0.10-0.50)
>500 to 500	0.95 (0.62-1.16)
500 to >350	3.00 (2.83-3.16)
350 to >200	3.74 (3.48-4.00)
>200 to 50	1.50 (1.13-2.25)
200 to onset of AIDS	~2.00 (1.50-3.00)

See Optima HIV User Guide for supporting information

The figure below illustrates the PLHIV on ART and their subsequent CD4 count in the baseline versus the no ART for 6-month scenario in 2020 for an example country. For ART disruption, this is for the default "off ART" mortality rate, CD4 counts dropped even more dramatically following the parameter informed by the SMART Study (1), as used herein.

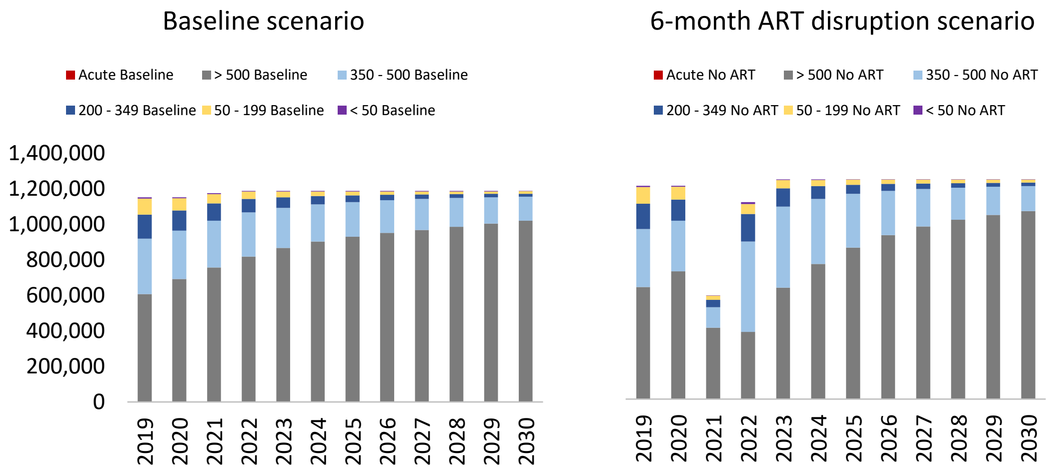

Figure A1. Distribution of CD4 count for people living with HIV both on and off ART (left panel) compared with a six-month complete of ART disruption scenario (right panel)

The default off ART mortality rate (see table A2) was applied for generating CD4 counts shown in figure A1; however, mortality rate off ART used in this study followed SMART Study findings, which would have showed even more dramatic CD4 reductions particularly in 2021.

Figure A2 illustrates the difference in progression of CD4 counts among adults living with HIV either on or off ART for an example country in sub-Saharan Africa.

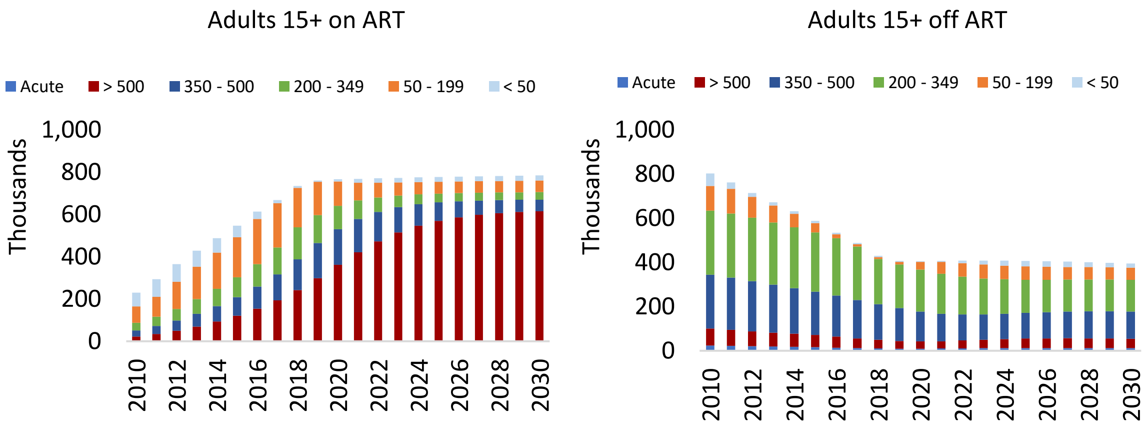

Figure A2. Distribution of estimated CD4 counts for adults aged 15 years and over who are living with HIV on ART (left panel) and off ART (right panel) for an example country in sub-Saharan Africa

3. Mortality rates

The HIV-related death rate for people living with HIV who were on ART, but who stopped treatment due to potential disruption on the antiretroviral (ARV) drug supply as a result of the COVID-19 pandemic was modeled to represent findings from the SMART Study (1). The SMART trial showed that immune recovery during ARV therapy tended to be rapidly lost after interruption of treatment, with a median loss of 187 CD4 cells/mm^3 and 25% of people experiencing a loss of greater than 317 cells/mm^3 in 2 months. HIV-death rates used in the Optima HIV model are listed in table A2 and figure A3.

Table A5. HIV-related death rates (percent mortality per year with low and high bounds) by CD4 stage or ART infection status

Stage or status	Best (low-high)
Acute infection	0.36% (0.29%-0.44%)
CD4 >500	0.36% (0.29%-0.44%)
CD4 350 to <500	0.58% (0.48%-0.71%)
CD4 200 to <350	0.88% (0.75%-1.01%)
CD4 50 to <200	5.90% (5.40%-7.90%)
CD4<50	32.30% (29.60%-43.20%)
Relative mortality rate on suppressive ART	23.00% (15.00%-30.00%)
Relative mortality rate off suppressive ART*	48.78% (28.35%-84.17%)

*With increased death rates for people on ART who stop ART as described above following SMART Study (1)
See Optima HIV User Guide Vol. VI: Parameter Data Sources for supporting information

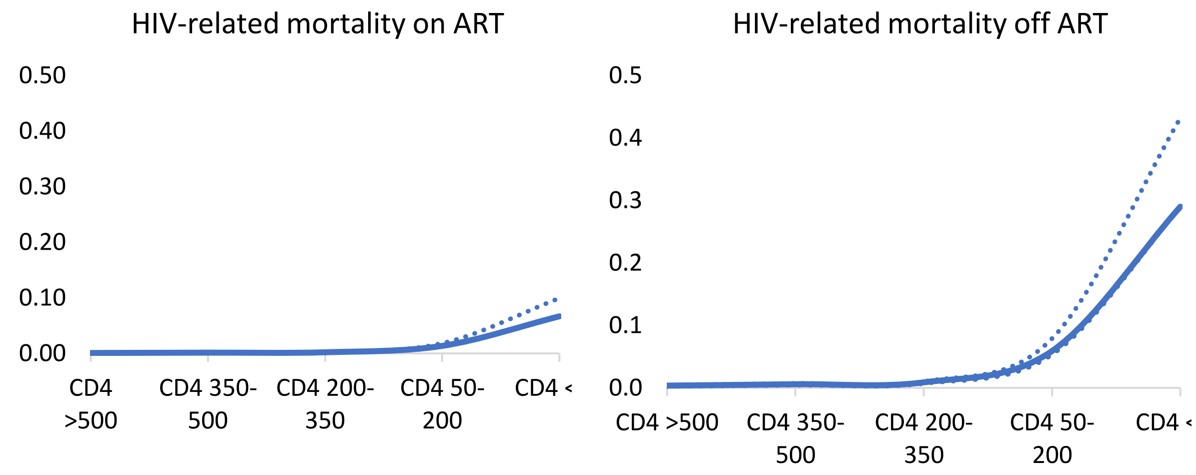

Figure A3. HIV-related mortality rates with bounds (area between dotted lines) by CD4 count for on and off ART used in the Optima HIV model

References

1. Grund B for the SMART Study Group. Predictors of initial CD4 decline after antiretroviral treatment interruption in the SMART study. Abstract THPE0144. XVI International AIDS Conference, 13-18 August 2006.

HIV Synthesis Model

Table A6. Description of setting scenarios at start of 2020 (n=300).

Characteristic	Model (2020) (median, 90% range)	Examples of observed data*
HIV prevalence (age 15-49)	9% (5% - 23%)	Zimbabwe 2016 13%, Tanzania 2017 5%, Uganda 2017 6%, Lesotho 2017 24%, Eswatini 2017 27%, Malawi 2016 10%, Namibia 2017 12%, Zambia 206 11%, Cameroon 2017 3.4%, Cote d'Ivoire 2017/18 2.5%, Mozambique 11%
HIV incidence age 15-49 (/100 person years)	0.51 (0.11 – 1.66)	Malawi 2016 0.37, Zambia 2016 0.66, Zimbabwe 2016 0.45, Lesotho 2017 1.55, Namibia 2016 0.40, Eswatini 2017 1.48, Tanzania 2017 0.27, Cameroon 2017 0.27
Proportion of men circumcised	0.34 (0.13 – 0.53)	Tanzania 2016 0.79 Lesotho 2016 0.68 South Africa 0.58 Uganda 2016 0.43 Namibia 2016 0.39 Eswatini 2016 0.30 Zambia 2016 0.28 Malawi 2016 0.25 Zimbabwe 2016 0.14
Percent of women that are sex workers	1.8% (0.4% -4.8%)	1.2% Zimbabwe (Fearon) 1%-4% Vandepitte et al.
Percent of people on PrEP	0.06% (0.01% - 0.22%)	
Proportion of HIV positive people diagnosed	93% (76% - 96%) (89% M, 95% W)	Malawi 2016 77%, Zambia 2016 67%, Zimbabwe 2016 74%, Namibia 2017 86%, Tanzania 2017 52%, Ethiopia 2018 72%, Cote d'Ivoire 2017/18 37%, Cameroon 2017 47%
Proportion of diagnosed HIV+ people on ART	94% (84% - 97%)	Lesotho 2016/17 92%, South Africa 2017 71%, Eswatini 2016/17 87%, Namibia 2017 96%, Zambia 2016 87%, Tanzania 2016/17 94%, Ethiopia 99%, Malawi 2016 91%, Uganda 2016/17 90%, Cameroon 2017 91%, Zimbabwe 2016 87%, Cote d'Ivoire 2017/18 88%, Cameroon 2017 91%.
Proportion of all HIV positive people with VL < 1000 copies/mL	68% (47% - 83%)	Zambia 2016 60%, Malawi 2016 68%, Zimbabwe 2016 60%, Eswatini 2017 73%, Lesotho 2017 68%, Tanzania 2017 52%, Uganda 2017 60%, Namibia 2017 77%, Ethiopia 2018 70%, Cote d'Ivoire 2017/18 40%, Cameroon 2017 47%.
Proportion of ART experienced people who have started 2nd-line ART	16% (2% - 39%)	Malawi ~3%% (Malawi MoH Quarterly Reports)
Of people on ART, proportion with VL < 1000	84% (62% - 93%) (80% M, 86% W)	Zambia 2016 Men/Women 88%/90%, Malawi 2016 90%/92%, Zimbabwe 2016 84%/88%, Namibia 2017 92%/90%, Tanzania 2017 89%/83%, Ethiopia 2018 95%/87%, Cote d'Ivoire 2017/18 76%, Cameroon 2017 80%.
Of people on ART, proportion with CD4 < 500 / mm^3	47% (39% - 59%)	Eswatini 40% 2016/17, Malawi 52% 2016, Tanzania 2017/17 55%, Zambia 2016 59%.
Of people on ART, proportion with CD4 < 200 / mm^3	9% (5% - 17%)	

Mother to child transmission rate	9% (0% - 27%)	(including breastfeeding period) Botswana 5%, South Africa 5%, Namibia 6%, Uganda 8%, Zimbabwe 7%, Malawi 9%, Tanzania 12%, Ethiopia 21%, Cote d'Ivoire 16%, Cameroon 15% (UNAIDS 2019)
Proportion of women age 15-65 giving birth per year	8% (3% - 18%)	South Africa 6%, Botswana 7%, Namibia 10%, Malawi 15%, Zimbabwe 12%, Uganda 18%, Tanzania 16%, Ethiopia 12%, , Cote d'Ivoire 15%, Cameroon 14%. (https://population.un.org/wpp/)

* all data from PHIA surveys (Population Health Impact Surveys. https://phia.icap.columbia.edu/), South Africa (HSRC survey), Botswana (BCPP 2013-15) unless stated.

Table A7. Amongst setting scenarios with all ART stopped, relative increase in HIV deaths according to proportion of people with HIV with VL < 1000 copies/mL

**Proportion of people with HIV with VL < 1000 copies/mL**	**Median relative increase in annual HIV-related deaths due to 6 mth disruption in 20% / 50% / 100% of people over 1 year.**
< 0.5	1.21 (1.13 – 1.31)<br>1.61 (1.37 – 1.76)<br>2.60 (1.87 – 3.09)
0.50-	1.22 (1.16 – 1.31)<br>1.64 (1.46 – 1.97)<br>2.70 (2.13 – 3.88)
0.55-	1.23 (1.11 – 1.31)<br>1.81 (1.30 – 1.96)<br>2.83 (1.69 – 3.85)
0.60-	1.27 (1.15 – 1.37)<br>1.81 (1.40 – 2.18)<br>3.26 (1.97 – 4.76)
0.65-	1.28 (1.13 – 1.36)<br>1.85 (1.35 – 2.14)<br>3.43 (1.82 – 4.59)
0.70-	1.30 (1.17 – 1.40)<br>1.94 (1.49 – 2.33)<br>3.78 (2.23 – 5.45)
0.75-	1.35 (1.18 – 1.49)<br>2.10 (1.52– 2.70)<br>4.43 (2.31 – 7.29)
0.80-	1.37 (1.26 – 1.47)<br>2.21 (1.78 – 2.64)<br>4.87 (3.18 – 6.95)

Table A8. Predicted absolute number of excess HIV deaths in 1 year due to 6 months interruption of ART taking in example countries

Country	Number (age 15+) living with HIV*	Proportion of people with HIV with VL < 1000*	Assumed proportion of people with HIV with VL < 1000 in 2020	Number of HIV deaths in adults age 15+ in 2018*	Predicted number of excess HIV deaths in adults in one year from 2020.5 due to interruption of ART taking for 6 months by 20% / 50% 100% of people on ART, due to disruption caused by COVID-19
**Malawi**	970,000	72%	70-75%<br>1.30 (1.17 – 1.40)<br>1.94 (1.49 – 2.33)<br>3.78 (2.23 – 5.45)	10,500	3,200 (1,800 – 4,200)<br>9,900 (6,100 – 14,000)<br>29,200 (12,900 – 46,700)
**Zimbabwe**	1,220,000	60%**	60-65%<br>1.27 (1.15 – 1.37)<br>1.81 (1.40 – 2.18)<br>3.26 (1.97 – 4.76)	18,700	5,000 (2,800 – 6,900)<br>15,100 (7,500 – 22,100)<br>42,300 (18,100 – 70,300)
**South Africa**	7,500,000	54%	50-55%<br>1.22 (1.16 – 1.31)<br>1.64 (1.46 – 1.97)<br>2.70 (2.13 – 3.88)	66,000	14,500 (10,600 – 20,500)<br>42,200 (30,400 – 64,000)<br>112,000 (74,600 – 190,000)
**Lesotho**	320,000	56%	55-60%<br>1.23 (1.11 – 1.31)<br>1.81 (1.30 – 1.96)<br>2.83 (1.69 – 3.85)	5,400	1,200 (600 – 1,700)<br>4,400 (1,600 – 5,200)<br>9,900 (3,700 – 15,400)
**Eswatini**	192,000	83%	80-85%<br>1.37 (1.26 – 1.47)<br>2.21 (1.78 – 2.64)<br>4.87 (3.18 – 6.95)	2,130	770 (470 – 970)<br>2,570 (1,670 – 3,470)<br>8,270 (4,670 – 12,670)
**Uganda**	1,280,000	65%	65-70%<br>1.28 (1.13 – 1.36)<br>1.85 (1.35 – 2.14)<br>3.43 (1.82 – 4.59)	18,000	2,300 (2,300 – 6,500)<br>15,300 (6,300 – 20,500)<br>43,700 (14,800 – 64,600)
**Mozambique**	2,000,000	assume < 50%	< 50%<br>1.21 (1.13 – 1.31)<br>1.61 (1.37 – 1.76)<br>2.60 (1.87 – 3.09)	45,000	9,500 (5,900 – 14,000)<br>27,500 (16,700 – 34,200)<br>72,000 (39,200 – 94,000)
**Kenya**	1,440,000	assume 70-75%	70-75%<br>1.30 (1.17 – 1.40)<br>1.94 (1.49 – 2.33)<br>3.78 (2.23 – 5.45)	20,100	6,000 (3,400 – 8,040)<br>18,900 (9,800 – 26,700)<br>55,900 (24,700 – 89,400)
**Tanzania**	1,460,000	63%	65-70%	18,600	5,200 (2,400 – 6,700)

			1.28 (1.13 – 1.36)<br>1.85 (1.35 – 2.14)<br>3.43 (1.82 – 4.59)		15,800 (6,500 – 21,200)<br>45,200 (15,300 – 66,800)
**Botswana**	350,000	83%	80%-85%<br>1.37 (1.26 – 1.47)<br>2.21 (1.78 – 2.64)<br>4.87 (3.18 – 6.95)	4,500	1,700 (1,200 – 2,100)<br>5,400 (3,500 – 7,400)<br>17,400 (9,800 – 26,800)
**Cameroon**	500,000	assume < 50%	< 50%<br>1.21 (1.13 – 1.31)<br>1.61 (1.37 – 1.76)<br>2.60 (1.87 – 3.09)	14,300	3,300 (1,900 – 4,400)<br>8,700 (5,300 – 10,900)<br>22,900 (12,400 – 29,900)
**Cote d'Ivoire**	430,000	< 50%	< 50%<br>1.21 (1.13 – 1.31)<br>1.61 (1.37 – 1.76)<br>2.60 (1.87 – 3.09)	13,700	2,900 (1,800 – 4,200)<br>8,400 (5,100 – 10,400)<br>21,900 (11,900 – 28,600)
**Nigeria**	1,770,000	< 50%	< 50%<br>1.21 (1.13 – 1.31)<br>1.61 (1.37 – 1.76)<br>2.60 (1.87 – 3.09)	40,000	8,400 (5,200 – 12,400)<br>24,400 (14,800 – 30,400)<br>64,000 (34,800 – 83,600)
**Western & Central Africa**	5,000,000	39%	< 50%<br>1.21 (1.13 – 1.31)<br>1.61 (1.37 – 1.76)<br>2.60 (1.87 – 3.09)	160,000	33,600 (20,800 – 49,600)<br>97,600 (59,200 – 121,600)<br>256,000 (139,200 – 334,400)
**Eastern & Southern Africa**	20,600,000	58%	60-65%<br>1.27 (1.15 – 1.37)<br>1.81 (1.40 – 2.18)<br>3.26 (1.97 – 4.76)	310,000	83,700 (46,500 – 114,700)<br>251,100 (124,000 – 365,800)<br>700,600 (300,700 – 1,165,600)
**Sub Saharan Africa**	25,600,000	(5000000 x .39 + 20,600,000 x 0.58) / 25,600,000 = 54%	55-60%<br>1.23 (1.11 – 1.31)<br>1.81 (1.30 – 1.96)<br>2.83 (1.69 – 3.85)	470,000	108,100 (51,700 – 145,700)<br>380,700 (141,000 – 451,200)<br>860,100 (324,300 – 1,339,500)

* UNAIDS estimates; ** PHIA 2016

Reference

1. Phillips AN, et al. Updated assessment of risks and benefits of dolutegravir versus efavirenz in new antiretroviral treatment initiators in sub-Saharan Africa: modelling to inform treatment guidelines. Lancet HIV 2020. https://www.thelancet.com/journals/lanhiv/article/PIIS2352-3018(19)30400-X/fulltext

   Appendix: https://www.thelancet.com/cms/10.1016/S2352-3018(19)30400-X/attachment/3398d3d8-5988-4171-aa74-2f68ce877725/mmc1.pdf

Modelling of effects of interruption of ART in HIV Synthesis

This below is from the appendix to reference 1 describing the Viral load and CD4 count changes during ART interruption. https://www.thelancet.com/cms/10.1016/S2352-3018(19)30400-X/attachment/3398d3d8-5988-4171-aa74-2f68ce877725/mmc1.pdf Risk of AIDS death is related to the most recent CD4 count, viral load and age, as described in that appendix.

Viral load returns to previous maximum viral load (vmax) in 3 months and adopts natural history changes thereafter.

CD4 rate of decline returns to natural history changes (i.e. those in ART naïve patients) after 9 months, unless the count remains > 200 above the CD4 nadir

Rate of CD4 count decline depends on current viral load. c(t) is the CD4 count at time t, cmin(t) is the CD4 count nadir measured by time t and cc(t-1) is the change in CD4 count from t-1 to t.

if time off ART = 3 months or if time off ART > 3 months and CD4 in previous period is > 300 above the minimum CD4 count to date

v(t) = vmax(t-1)

if v(t) $\geq$ 5	then cc(t-1) = Normal $(-200,10^2)$
if 4.5 <= v(t) < 5	then cc(t-1) = Normal $(-160,10^2)$
if v(t) < 4.5	then cc(t-1) = Normal $(-120,10^2)$

If this leads to c(t) < cmin(t) (CD4 nadir) then c(t) is set to cmin(t)

if time off ART = 6 months:-

if v(t) $\geq$ 5	then cc(t-1) = Normal $(-100,10^2)$
if 4.5 <= v(t) < 5	then cc(t-1) = Normal $(-90,10^2)$
if v(t) < 4.5	then cc(t-1) = Normal $(-80,10^2)$

if time off ART = 9 months:-

if v(t) $\geq$ 5	then cc(t-1) = Normal $(-80,10^2)$
if 4.5 <= v(t) < 5	then cc(t-1) = Normal $(-70,10^2)$
if v(t) < 4.5	then cc(t-1) = Normal $(-60,10^2)$

This is broadly based on evidence from a number of analyses of the effects of ART interruption (1-5 below)

1. d'Arminio Monforte A, Cozzi Lepri A, Phillips AN, et al. Interruption of HAART in HIV clinical practice. Results from the ICONA study. JAIDS 2005; 38: 407-416
2. Li X, Margolick JB, Conover CS, et al. Interruption and discontinuation of HART in the MACS. JAIDS 2005; 38: 3:320-328.
3. Mocroft A, Youle M, Moore A, et al. Reasons for modification and discontinuation of antiretrovirals: results from a single treatment centre. AIDS 2001; 15 (2): 185-194.
4. Wit FWNM, Blanckenberg DH, Brinkman K, et al. Safety of long-term interruption of successful antiretroviral therapy: the ATHENA cohort study. AIDS 2005; 19: 345-348.
5. Grund B for the SMART Study Group. Predictors of initial CD4 decline after antiretroviral treatment interruption in the SMART study. XVI International AIDS Conference. August 13-18, 2006. Toronto. Abstract THPE0144.

Imperial College London Model

The Imperial College London Model is a deterministic, compartmental model of HIV transmission and progression, and has been described previously [1,2]. Briefly, the model is stratified by age, sex, behavioural risk group (including partner change rate, condom use, and frequency of sex acts), and male circumcision status, with population growth in accordance with country-specific projections. HIV-infected individuals in the model progress through HIV infection based on stage of infection, CD4 count, and antiretroviral therapy (ART) status. Rates of transmission within the model are based on the risk group of both partners, infection stage and ART status of the HIV-positive partner, and the circumcision status of an HIV-negative male partner.

The model is calibrated over time to age and sex-specific HIV prevalence, number and proportion of HIV-infected individuals on ART, and male circumcision scale-up. Parameters fit in the calibration include the HIV transmission probability per sex act, sexual contact rates by behavioural risk group, proportion of the population in each risk group, rates of mixing between risk groups, and the start time of the epidemic. Analyses provided are for models calibrated for Malawi, South Africa, and Zimbabwe. The model's fit to adult HIV prevalence and proportion on ART in South Africa are shown in Supplementary Figure 1.

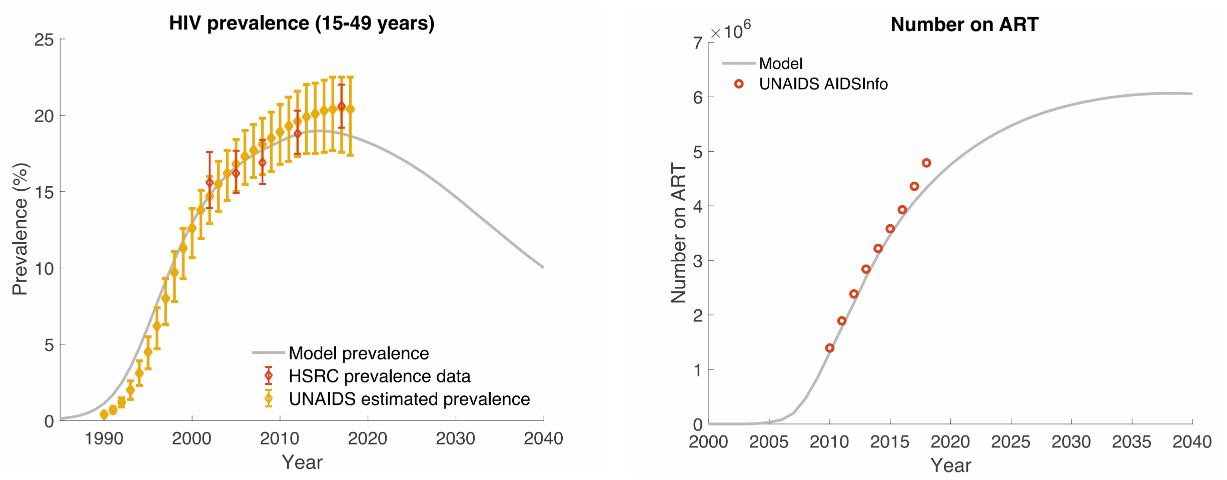

Figure A4: Modelled HIV prevalence and number on ART in the Imperial College London South Africa model.

This analysis simulates the effect of 3- and 6-month interruptions of different HIV services starting mid-2020 as a result of the COVID-19 pandemic. In all scenarios of interruptions to HIV services, there is assumed to be no change to sexual contact rates, but a scenario of a 10% reduction in sexual contacts for all risk groups is modelled separately. Each type of disruption is assumed to occur independently so as to illustrate the individual effect of the respective disruption. However, in reality, multiple disruptions might occur simultaneously.

Results are most sensitive to the assumption about mortality for HIV-infected individuals who stop ART. Our results are therefore presented using three different estimates of mortality, with the central result using the 'medium' assumption in Supplementary Table 1.

	Average monthly mortality risk	*Proportion that would die after one year*	*Justification*
Lower bound	0.10%	1.24%	This is a hypothetical best-case scenario in which the vast majority of individuals do not deteriorate rapidly.
Medium	0.24%	2.91%	The SMART trial found a 3% risk at 12 months of either death of an opportunistic infection for those with interrupted ART [3]. This also implies a mean survival time approximately equivalent to that for HIV-positive persons who have never been on ART [4].
Upper bound	0.44%	5.28%	This is the hypothetical worst-case scenario in which many persons deteriorate more rapidly.

Table A9: Alternative assumptions used for the risk of death experienced by those PLHIV for whom their ART supply is interrupted.

1. Cremin, I., et al., *The new role of antiretrovirals in combination HIV prevention: a mathematical modelling analysis.* AIDS, 2013. **27**(3): p. 447-458.
2. Smith, J.A., et al., *Maximising HIV prevention by balancing the opportunities of today with the promises of tomorrow: a modelling study.* The Lancet HIV, 2016. **3**(7): p. e289-e296.
3. The Strategies for Management of Antiretroviral Therapy (SMART) Study Group, *CD4+ Count–Guided Interruption of Antiretroviral Treatment.* New England Journal of Medicine, 2006. **355**(22): p. 2283-2296.
4. Todd, J., et al., *Time from HIV seroconversion to death: a collaborative analysis of eight studies in six low and middle-income countries before highly active antiretroviral therapy.* AIDS, 2007. **21**(Suppl 6): p. S55-63.

EMOD HIV Model

The EMOD model source code, parameter definitions and ranges, and description/documentation can be found at https://github.com/InstituteForDiseaseModeling/EMOD. Analyses are based upon a model calibrated to the HIV epidemic in South Africa and described elsewhere.[1-4] Briefly, the model is parameterized with epidemiologic data including population size, fertility, mortality, voluntary male circumcision coverage, health seeking and sexual behaviour. South Africa data on age and sex specific HIV prevalence, ART coverage, population size and HIV incidence were used to calibrate the model. Calibration was performed using a parallel simultaneous perturbation optimization (PSPO) algorithm. [5] Roulette resampling in proportion to the likelihood of each simulation was used to select 250 model parameter sets. The model's fit to adult HIV prevalence and number receiving ART are shown in Figure A5 (gray lines: individual simulations; blue line: average of 250 simulations; black dots/lines: data from HIV surveys).

Figure A5.

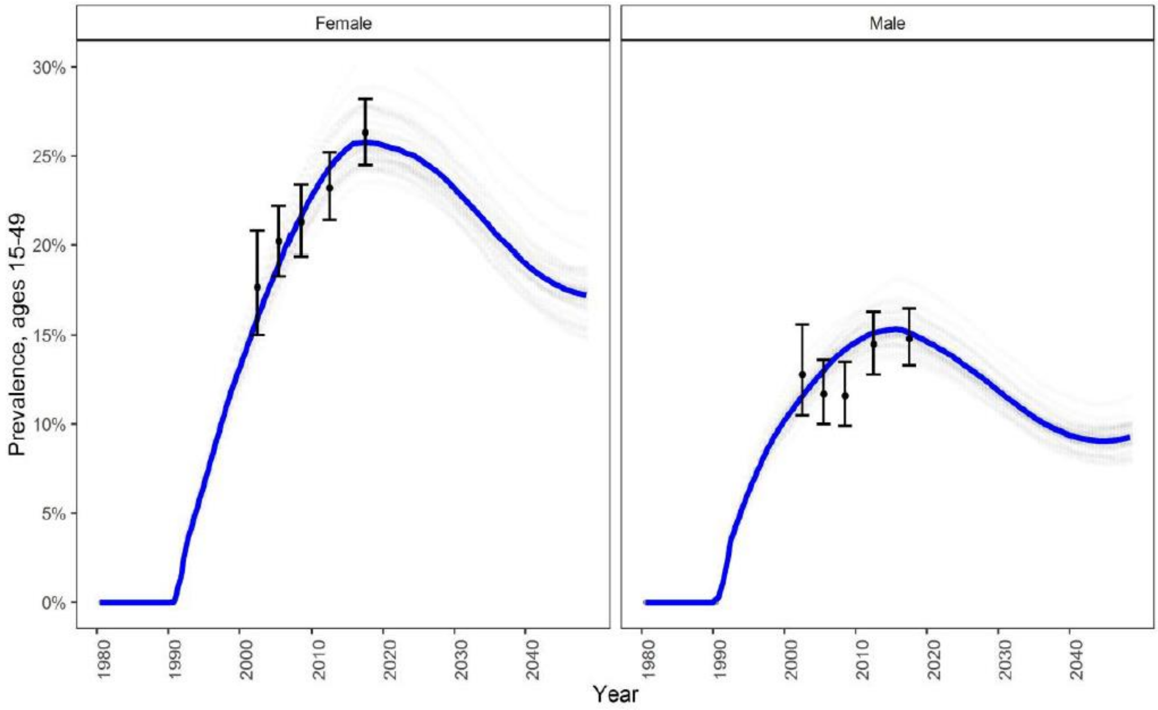

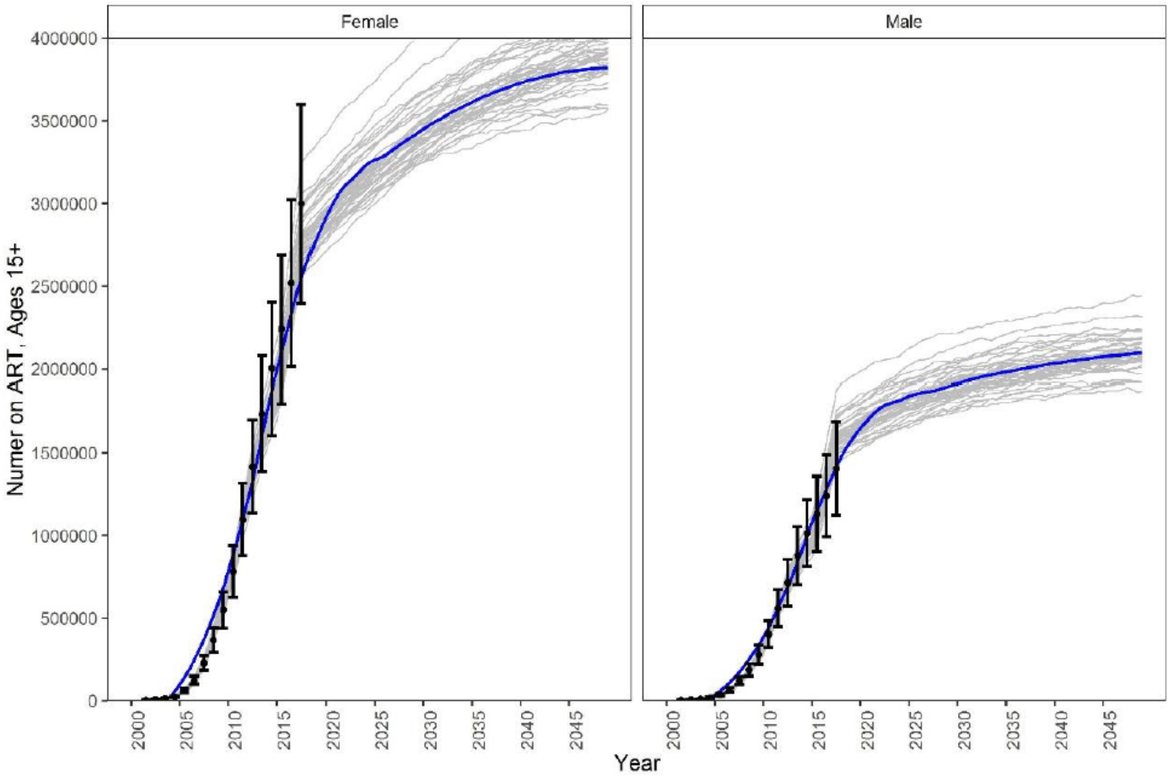

To estimate the number of excess deaths in countries and regions throughout sub-Saharan Africa, we assumed that the number of deaths caused by a 6-month interruption in ART was proportional to the number of people virally suppressed on ART in a given country or region. A limitation of this assumption is that it does not take into account regional differences in the vulnerability of PLHIV with viral load suppression to death from an ART interruption, which could arise due to differences in the age distribution, CD4 count, health and nutritional status, and exposure to infections such as tuberculosis. Data used to estimate mortality by country and region are shown in Supplementary Table 1

Table A10. Estimation of excess HIV deaths in 1 year due to 6 months interruption of ART with 95% uncertainty bounds.

Country	Number of PLHIV[a]	Number of people receiving ART[a]	Percent of people on ART with Viral Load Suppression (95% CI)	Predicted number of excess HIV deaths in adults in one year from 2020.5 due to interruption of ART taking for 6 months due to disruption caused by COVID-19 (95% CI)
South Africa	7,700,000	4,788,000	54% (49 - 58)	138,126 (35,404 – 4,850)
Malawi	1,000,000	814,000	69% (62 – 75)	22,921 (19,360 – 27,406)
Mozambique	2,200,000	1,213,000	----	
Zimbabwe	1,300,000	1,155,251	59.6% (57.4 -61.8)[b]	25,738 (20,975 – 30,794)
Eswatini	210,000	177,000	81% (76 -89)	5,651 (4,797 – 6,504)
Lesotho	340,000	206,000	57% (53 – 60)	6,438 (5,634 – 7,175)
Uganda	1,400,000	1,004,000	64% (60 – 69(	29,765 (25,911 – 34,382)
Kenya	1,600,000	1,068,000	72%(69 – 74)[c]	38,056 (29,711 – 46,959)
Botswana	370,000	307,000	81% (73 – 88)	9,956 (8,003 – 11,693)
Tanzania	1,600,000	1,109,000	62% (56 – 68)	32,954 (26,044 – 38,402)

East and Southern Africa	20,600,000	13,802,000	58% (50 – 66)	138,126 (35,404 – 4,850)
				22,921 (19,360 – 27,406)
Cameroon	540,000	281,000	79% (78- 82)[d]	
Côte d'Ivoire	460,000	252,000	41% (33 – 52)	25,738 (20,975 – 30,794)
Nigeria	1,900,000	1,016,000	42% (32 – 57)	5,651 (4,797 – 6,504)
West and Central Africa	5,000,000	2,550,000	39% (25 -53)	6,438 (5,634 – 7,175)

[a] UNAIDS estimate for the most recent available year (2018 or 2019) obtained from http://aidsinfo.unaids.org/

[b] Estimate from the 2016 Zimbabwe Population HIV Impact Assessment (ZIMPHIA), https://phia.icap.columbia.edu/countries/zimbabwe/

[c] Estimate from the Preliminary Report of the 2018-19 Kenya Population HIV Impact Assessment (KENPHIA), https://phia.icap.columbia.edu/countries/kenya/

[d] Estimate from: Fokam, J., Sosso, S.M., Yagai, B. et al. Viral suppression in adults, adolescents and children receiving antiretroviral therapy in Cameroon: adolescents at high risk of virological failure in the era of “test and treat”. AIDS Res Ther 16, 36 (2019). https://doi.org/10.1186/s12981-019-0252-0

References

1. Bershteyn A, Gerardin J, et al., “Implementation and applications of EMOD, an individual-based multi-disease modeling platform", *Pathogens and Disease*, Volume 76, Issue 5, July 2018, fty059, https://doi.org/10.1093/femspd/fty059

2. Akullian A, Bershteyn A, Klein D, Vandormael A, Bärnighausen T, Tanser F. Sexual partnership age pairings and risk of HIV acquisition in rural South Africa. *AIDS*. 2017;31(12):1755-1764. doi:10.1097/QAD.0000000000001553

3. Selinger S, Bershteyn A, et al. Targeting and vaccine durability are key for population-level impact and cost-effectiveness of a pox-protein HIV vaccine regimen in South Africa, Vaccine, Volume 37, Issue 16, 2019, Pages 2258-2267, https://doi.org/10.1016/j.vaccine.2019.02.073.

4. Selinger C, Dimitrov DT. *et al.* The future of a partially effective HIV vaccine: assessing limitations at the population level. *Int J Public Health* **64,** 957–964 (2019). https://doi.org/10.1007/s00038-019-01234-z

5. Alaeddini A and Klein DJ. Application of a second-order stochastic optimization algorithm for fitting stochastic epidemiological models. In Proceedings of the 2017 Winter Simulation Conference (WSC ’17). IEEE Press, Article 174, 1–12 (2017).
